# In vitro fertilization induces reproductive changes in male mouse offspring and has multigenerational effects

**DOI:** 10.1172/jci.insight.188931

**Published:** 2025-03-04

**Authors:** Eric A. Rhon-Calderon, Cassidy N. Hemphill, Alexandra J. Savage, Laren Riesche, Richard M. Schultz, Marisa S. Bartolomei

**Affiliations:** 1 Epigenetics Institute, Department of Cell and Developmental Biology, Perelman School of Medicine, University of Pennsylvania, Philadelphia, Pennsylvania, USA.; 2 Department of Biology, School of Arts and Sciences, University of Pennsylvania, Philadelphia, Pennsylvania, USA.; 3 Department of Microbiology and Molecular Genetics, University of California, Davis, Davis, California, USA.; 4 Center for Women’s Health and Reproductive Medicine, Perelman School of Medicine, University of Pennsylvania, Philadelphia, Pennsylvania, USA.

**Keywords:** Development, Reproductive biology, Epigenetics, Fertility, Mouse models

## Abstract

In vitro fertilization (IVF) is a noncoital method of conception used to treat human infertility. Although IVF is viewed as largely safe, it is associated with adverse outcomes in the fetus, placenta, and adult offspring. Because studies focusing on the effect of IVF on the male reproductive system are limited, we used a mouse model to assess the morphological and molecular effects of IVF on male offspring. We evaluated 3 developmental stages: 18.5-day fetuses and 12- and 39-week-old adults. Regardless of age, we observed changes in testicular-to-body weight ratios, serum testosterone levels, testicular morphology, gene expression, and DNA methylation. Also, sperm showed changes in morphology and DNA methylation. To assess multigenerational phenotypes, we mated IVF-conceived and naturally conceived males with wild-type females. Offspring from IVF males exhibited decreased fetal-to-placental weight ratios and changes in placenta gene expression and morphology regardless of sex. At 12 weeks of age, offspring showed higher body weights and differences in glucose, triglyceride, insulin, total cholesterol, HDL-C, and LDL/VLDL-C levels. Both sexes showed changes in gene expression in liver, testes, and ovaries and decreased global DNA methylation. Collectively, our findings demonstrate that male IVF offspring exhibit abnormal testicular and sperm morphology and molecular alterations with a multigenerational impact.

## Abstract 2

Male IVF-offspring in mice exhibit morphological and molecular reproductive changes, including testicular abnormalities and altered gene expression, with multigenerational effects on growth and metabolism in both fetys and placenta.

## Graphical abstract

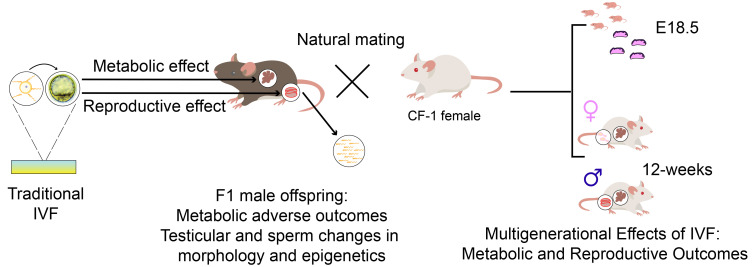

## Introduction

In vitro fertilization (IVF) is a widely used assisted reproductive technology (ART) to treat infertility (1). Since the first successful human IVF birth in 1978, this technology has dramatically evolved, resulting in improved pregnancy rates (2). Although considered safe, IVF pregnancies are associated with an increased risk of perinatal, neonatal, and placental complications; rare genetic syndromes; and possible long-term effects in human and mouse offspring (3). One possible mechanism for adverse outcomes suggests that IVF procedures occur during critical windows of epigenetic reprogramming in gametes and preimplantation embryos (3, 4), generating errors that could ultimately affect normal development. In fact, embryo culture is a major contributor to adverse IVF outcomes (5). Although IVF culture conditions have been modified to improve fertilization rates and blastocyst formation (6), no concomitant improvement in offspring outcomes has been observed. Similarly, techniques such as vitrification and preimplantation genetic testing have been integrated into standard IVF cycles, aiming to enhance pregnancy success (3, 7). As IVF becomes more widely used because of factors like delayed childbearing, same-sex couples seeking parenthood, and increasing incidence of infertility, understanding the potential adverse outcomes for the next generation becomes more crucial (8). This concern is furthered by an interest in identifying multigenerational and transgenerational changes due to IVF and other ART procedures (9).

The mouse has emerged as an excellent model to study ART, simulating the human counterpart and enabling more invasive studies (10). Previous studies using mouse models have shown that IVF procedures are associated with a higher risk of metabolic syndromes in offspring, which includes alterations in glucose, insulin, triglycerides, cholesterol, and the liver transcriptome and proteome compared with naturally conceived offspring (7, 11–19). Nevertheless, there is a paucity of information regarding potential effects on the reproductive system in ART-conceived offspring as well as the effects of traditional IVF on the next generation. Previous studies have shown that testicular or sperm maturation changes affect normal sperm function, which leads to adverse outcomes in sired offspring (20). A recent study using a mouse model demonstrated that metabolic changes in male IVF offspring, such as increased body weight, altered insulin levels, and changes in fat deposition, were transmitted to the second generation in the absence of an additional insult, such as a high-fat diet. However, the reproductive system was not examined, and the mechanism underlying the multigenerational effects was not pursued (21).

Intracytoplasmic sperm injection (ICSI) is widely used for male-specific infertility or fallopian tube obstruction (20). ICSI has similarities to conventional IVF, including embryo culture and transfer (22). Although men conceived via ICSI/IVF had similar sperm concentrations, total sperm counts, and total motile counts to those conceived naturally, subtle but significant differences were observed in sperm morphology and progressive motility, as well as in gonadotropin and serum testosterone levels (23). Another study, using mice, showed that male, ICSI-conceived offspring not only showed changes in behavior and DNA methylation at promoter regions in testis DNA but also reported a transgenerational effect of ICSI, including changes in behavior and testes’ DNA methylation levels (9).

Although numerous adverse changes to placentas, liver, and metabolic profiles have been observed in mice conceived by IVF (7, 11, 24, 25), to our knowledge, no study has focused specifically on the male reproductive system. To address this gap, we assessed the morphological and molecular effects of IVF on the testes and sperm of male offspring and then evaluated whether these adverse outcomes influenced the next generation. We report that IVF-conceived males exhibit changes in testicular-to-body weight ratios, decreased testosterone levels, alterations in seminiferous tubule morphology, DNA methylation perturbations in testes and sperm, and altered gene expression compared with naturally conceived offspring. Moreover, second-generation offspring display metabolic perturbations and transcriptome changes in liver, testis, and ovaries and changes in global DNA methylation of testes and ovaries.

## Results

### Effects of IVF on testis weight and histology.

**Figure 1:**
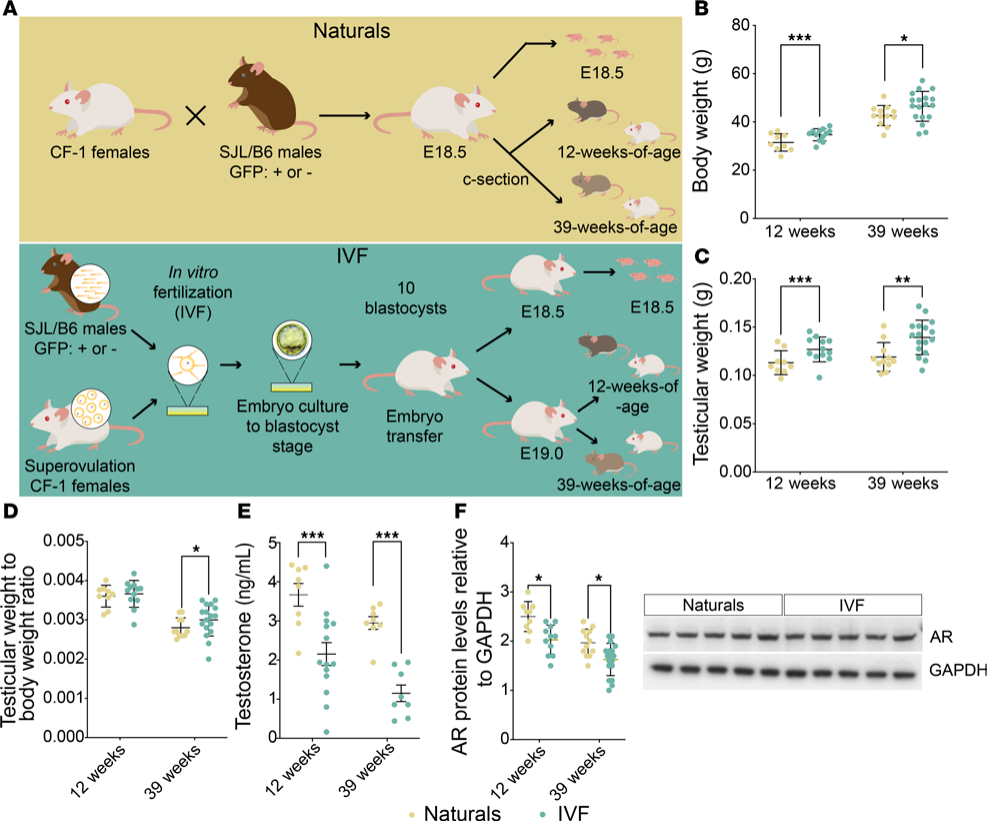
Mouse model and general parameters. (**A**) Mouse model: Top: Naturally conceived embryos (Naturals) were generated using SJL/B6 male mice and CF-1 female mice, and bottom: IVF embryos were produced using capacitated sperm from SJL/B6 GFP^+^ or GFP^–^ mice and eggs from superovulated CF-1 mice. Embryos were cultured to the blastocyst stage; 10 blastocysts were transferred to pseudopregnant females. At E18.5 pregnant females from both groups were C-sectioned, and fetuses were delivered and collected for molecular analysis. For adult cohorts C-section–delivered pups at E18.5 (Naturals) or E19.0 (IVF) were fostered with wild-type dams and monitored until 12 or 39 weeks of age. Shown are (**B**) body weights before 6 hours of fasting, (**C**) testicular weights, (**D**) testicular/body weight ratios, (**E**) serum testosterone levels assayed by ELISA, and (**F**) representative immunoblot testicular protein levels of AR relative to GAPDH assayed by Western blots. Data are depicted as mean ± SEM; *n* = 10–12 per group. The black line represents the mean of each group. Statistical significance was determined by *t* test; *P < 0.05, **P < 0.01, and ***P < 0.001 IVF groups compared with Naturals.

To ascertain whether IVF could influence male reproductive function, we first assessed the effect of IVF on testes’ weight and histology using our mouse model (Figure 1A). Mean body and testicular weight at both 12 and 39 weeks were significantly higher in offspring of the IVF group compared with naturally conceived controls (naturals) (Figure 1, B and C), which led to a significantly higher testicular-to-body weight ratio for the IVF group in 39-week offspring (Figure 1D). Because testes are the primary producers of the key male sex hormone, testosterone, they play a critical role in both physical and reproductive health (26). Due to testosterone’s effect on multiple systems, abnormal testosterone levels can be indicators of underlying testicular and overall adverse health conditions (27). Accordingly, we determined whether an increase in testicular weight correlated with changes in testosterone levels and expression of the androgen receptor (AR) and found that both testosterone levels and AR expression in testis were significantly reduced in the IVF group at both 12 and 39 weeks compared with naturals (Figure 1, E and F).

**Figure 2:**
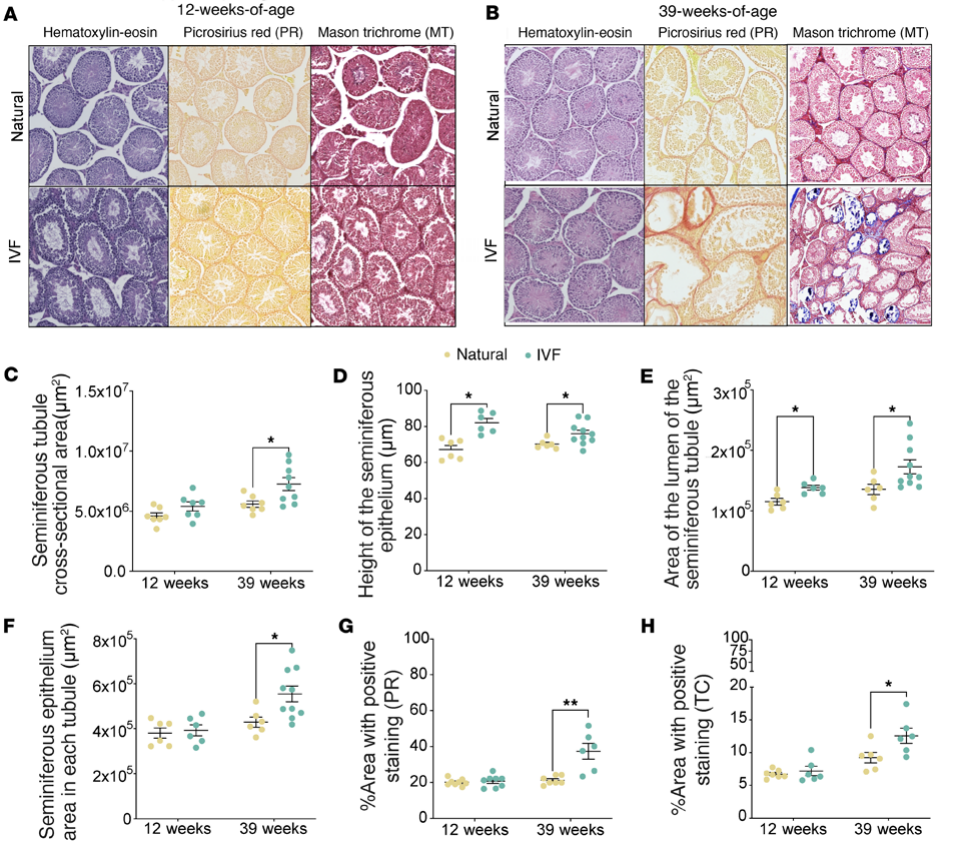
Testicular morphology analysis using hematoxylin-eosin, Picrosirius red, and Masson’s trichrome staining. Testicular cross sections at (**A**) 12 weeks of age and (**B**) 39 weeks of age using hematoxylin-eosin, Picrosirius red (PR), and Masson’s trichrome (MT). (Original magnification is 4×.) Morphology of the seminiferous tubules in hematoxylin-eosin slides, including (**C**) whole cross-sectional area, (**D**) height, (**E**) lumen area, and (**F**) total seminiferous epithelium area. Percentage of positively stained area with PR (**G**) and MT (TC) (**H**) is depicted. The black line represents the mean of each group. Each data point represents an individual conceptus from a minimum of 5 litters (*n* = 5–10/group). Data are expressed as mean ± SEM. Statistical analysis between groups was done by *t* test; *P < 0.05, and **P < 0.01, when comparing IVF against Naturals.

Spermatogenesis is a complex process involving the proliferation and differentiation of germ cells. Several factors could influence this process, including alterations in the seminiferous epithelium (28). Previous studies showed that seminiferous epithelium changes impair elongation of spermatids, affect axoneme formation, and disturb interactions between Sertoli cells and germ cells that lead to abnormal spermatogenesis (28). Seminiferous epithelium perturbations also arrest spermatogenesis at various developmental stages in mutant mice (29). Thus, any adverse changes to the seminiferous epithelium may impair spermatogenesis and affect male fertility. We therefore histologically examined the seminiferous epithelium at 12 and 39 weeks (Figure 2, A and B). Hematoxylin-eosin staining revealed increased seminiferous tubule cross-sectional area and seminiferous epithelium area per tubule at 39 weeks but not at 12 weeks (Figure 2, C and F), whereas the height and lumen of the seminiferous epithelium were increased in both 12- and 39-week offspring (Figure 2, D and E).

In testes, collagen, a major component of the testicular basement membrane, is located at the base of the seminiferous epithelium (30). The basement membrane has multiple functions, including germ cell movement and communication between different cell types (31). Disruption of this matrix is associated with azoospermia (32). Additionally, excessive collagen is associated with an increase in tunica albuginea weight and thickening of the basement membrane, as seen in fibrosis and aging models (30). We therefore assessed the effect of ART on the extracellular matrix in testis using 2 staining protocols: PR, which quickly and efficiently assesses collagen fiber content, organization, and orientation (33), and MT, which not only detects the collagen fiber deposition but also can distinguish muscle fibers (red), collagen (blue), and nuclei (black) simultaneously (34). Both methods revealed increased staining intensity exclusively at 39 weeks (Figure 2, G and H). To validate this increased staining, we assayed collagen type I alpha 1 chain (*Col1a1*) RNA and protein by reverse transcription quantitative PCR (RT-qPCR) and immunoblotting, respectively, as well as another extracellular matrix marker, vascular cell adhesion protein 1 (*Vcam1*), all of which were increased in IVF offspring compared with naturals (Supplemental Figure 1; supplemental material available online with this article; https://doi.org/10.1172/jci.insight.188931DS1). Finally, to determine whether changes in testicular size are due to alterations in cell composition — specifically spermatogonia, spermatocytes, and round spermatids — we performed an immunohistochemistry analysis of testicular sections using anti-SYCP3, PNA-488 for acrosome staining, and DAPI. At 12 weeks, we did not observe changes in cell composition in whole testis (Supplemental Figure 2, A and B). However, at 39 weeks, we observed a decrease in all cell types (Supplemental Figure 2, C and D), which could indicate an early loss of cells essential for spermatogenesis, potentially influencing future fertility. Taken together, these results indicate an increased thickening of the tubular walls and a greater degree of tubule disorganization with higher collagen content in IVF male offspring, with no significant changes in cell composition at 12 weeks but with changes by 39 weeks. Additionally, this increase in collagen deposition contributed to the increased testicular weight in IVF offspring.

### Effect of IVF on sperm count, morphology, and DNA methylation.

**Figure 3:**
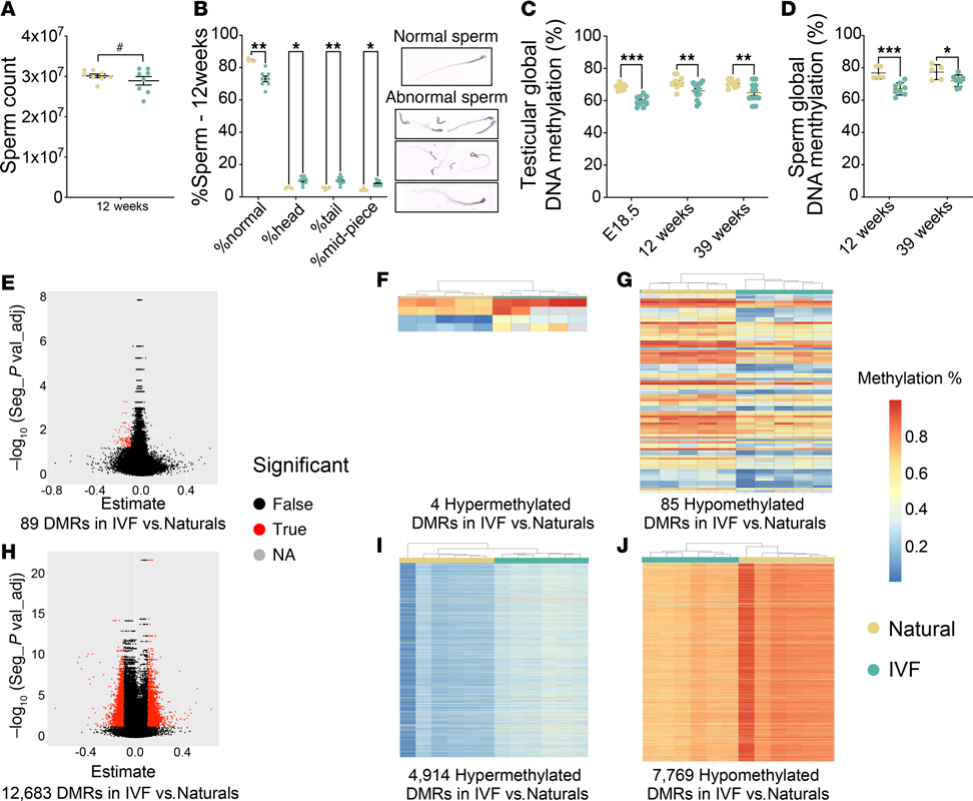
Sperm parameters and testicular DNA methylation. (**A**) Sperm count using a bright-field microscope; (**B**) sperm morphology quantification using eosin 1% staining where sperm is categorized as normal (%normal), abnormal head (%head), abnormal tail (%tail), and abnormal midpiece (%midpiece); (**C**) testicular global DNA methylation; and (**D**) sperm global DNA methylation using LUMA for 12- and 39-week natural and IVF offspring. Infinium Mouse Methylation BeadChip sperm analysis, including (**E**) volcano plot for DMRs at 12 weeks, 12 weeks heatmap with (**F**) hypermethylated DMRs and (**G**) hypomethylated DMRs, (**H**) volcano plot for DMRs at 39 weeks, and 39 weeks heatmap with (**I**) hypermethylated DMRs and (**J**) hypomethylated DMRs. Seg, segmentation analysis. Each data point represents 1 individual from a minimum of 5 litters (*n* = 10–15/group). Data are expressed as mean ± SEM. Statistical analysis between groups was done by *t* test; *, **, and *** represent significant differences of *P* < 0.05, *P* < 0.01, and *P* < 0.001 respectively, when comparing IVF and Naturals. Variability was calculated using an *F* test, and ^#^ represents a significant difference (*P* < 0.05) in IVF compared with Naturals.

Given the IVF-associated changes in testis morphology, we next examined the effect of IVF on several sperm parameters. Although there was no significant difference in sperm counts from 12-week-old IVF offspring, there was greater variability in sperm number (Figure 3A). When testes’ size and sperm count were correlated, IVF offspring with larger testes had lower sperm counts (Supplemental Figure 3), and the percentage of sperm with abnormal morphology was increased (Figure 3B). No data are available for the 39-week time point because sperm were immediately frozen, prohibiting characterization.

IVF is also associated with changes in whole-genome average DNA methylation (7, 11, 24, 25). We therefore assessed global DNA methylation using a luminometric methylation assay (LUMA) on testis DNA at E18.5, 12 weeks, and 39 weeks and sperm DNA at 12 and 39 weeks. We observed statistically significant decreased DNA methylation in testes and sperm from IVF-conceived male offspring compared with naturals at all time points (Figure 3, C and D). To identify specific regions with altered DNA methylation that could be transmitted to offspring, we examined DNA methylation in sperm from 12- and 39-week-old IVF-conceived mice using the Infinium Mouse Methylation-12v1-0 BeadChip array, which assays approximately 285,000 CpG probes distributed across the genome (7, 24, 35, 36). As previously described (7, 35), we first used all probes to determine the differentially methylated loci (DMLs) that had a 5% absolute change (0.05 difference) between groups. With these DMLs, we then determined differentially methylated regions (DMRs) that are associated with a differentially methylated probe on the array. Given that changes between 10% and 15% in methylation can influence gene expression and produce a phenotype (37), we considered a DMR a genomic region that had a 10% difference in methylation between groups. Heatmap and principal component analysis (PCA) by probe passing rate, group, and age showed a differential clustering between sperm from IVF and natural offspring (Supplemental Figure 4). At 12 weeks, we observed 89 DMRs, 4 hypermethylated DMRs, and 85 hypomethylated DMRs in IVF compared with naturals (Figure 3, E–G, and Supplemental Table 3). Gene ontology analysis failed to detect a significant association with specific biological functions at 12 weeks. Finally, in 39-week sperm samples, we observed that IVF-conceived males exhibited 12,683 DMRs, 4,914 hypermethylated DMRs, and 7,769 hypomethylated DMRs compared with naturals (Figure 3, H–J, and Supplemental Table 4). Gene ontology analysis showed that the most affected pathways related to cell-cell interactions, retrotransposon silencing, regulation of membrane potential, signal transduction, cell morphogenesis, and regulation of actin filament processes (Supplemental Figure 4E). Together these results show that IVF procedures impacted both testicular and sperm DNA methylation, with possible effects on the next generation, and that age increased the number of DMRs in sperm.

### Effect of IVF on testicular gene expression.

**Figure 4:**
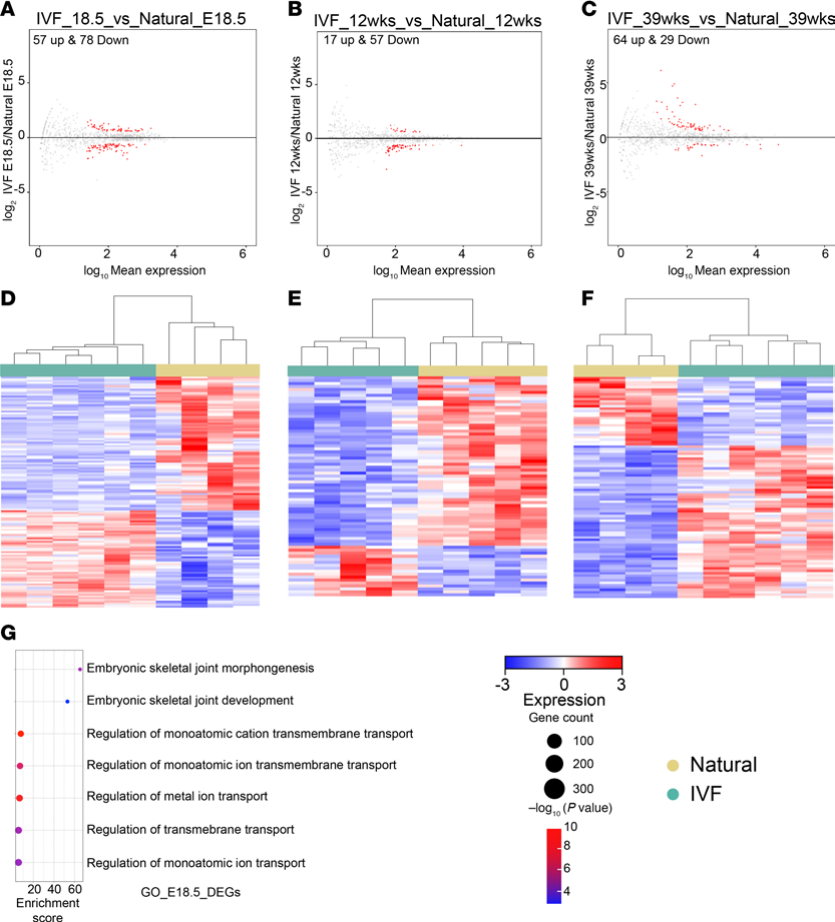
Transcriptome analysis of testicular tissues by RNA-Seq. Volcano plots showing differentially expressed genes (DEGs) for (**A**) E18.5 gonads and testis at (**B**) 12 weeks and (**C**) 39 weeks IVF versus natural. Heatmap for log_2_-transformed expression levels obtained from RNA-Seq of DEGs in (**D**) E18.5 gonads and testis at (**E**) 12 weeks and (**F**) 39 weeks. Gene ontology analysis for relevant affected pathways: (**G**) E18.5 testis. Color boxes at the top denote the experimental group.

To ascertain whether the changes in DNA methylation are associated with changes in gene expression of IVF-conceived males, we performed RNA-Seq on fetal and adult testes collected at E18.5, 12 weeks, and 39 weeks. PCA showed a clear separation between IVF and naturals (Supplemental Figure 5, A–C). Volcano plots revealed 57 upregulated and 78 downregulated genes at E18.5 (Figure 4A and Supplemental Table 5), 17 upregulated and 57 downregulated genes at 12 weeks (Figure 4B and Supplemental Table 6), and 64 upregulated and 29 downregulated genes at 39 weeks (Figure 4C and Supplemental Table 7). Not all DEGs at 12 weeks were observed at 39 weeks. Testes from IVF-conceived males at E18.5 had the greatest number of DEGs, and this difference did not appear to worsen with age. The heatmap for all DEGs showed tight clustering for all naturals, with IVF groups clustering together (Figure 4, D–F). Gene ontology analysis showed dysregulation in transmembrane transport and testicular function at E18.5 but failed to detect a significant association with specific biological functions at 12 and 39 weeks (Figure 4G). Finally, we determined if the DEGs in testis overlap with affected sperm DMRs. At 12 weeks, we did not observe any overlap between the DEGs and DMRs (Supplemental Figure 5D), whereas at 39 weeks, we observed that expression of 1 downregulated gene, *Ttc22*, and 8 upregulated genes, *1700019P21Rik*, *Cd24a*, *Gm31447*, *Nedd9*, *8430426J06Rik*, *Dazl*, *Sycp1*, and *Adamts9*, was linked with hypermethylated and hypomethylated DMRs, respectively (Supplemental Figure 5E). These data suggest little correlation between changes in gene expression and DNA methylation, indicating that the observed changes in gene expression may be due to epigenetic modifications not measured in this study.

### Multigenerational effects of IVF on placentas from F2 concepti.

**Figure 5:**
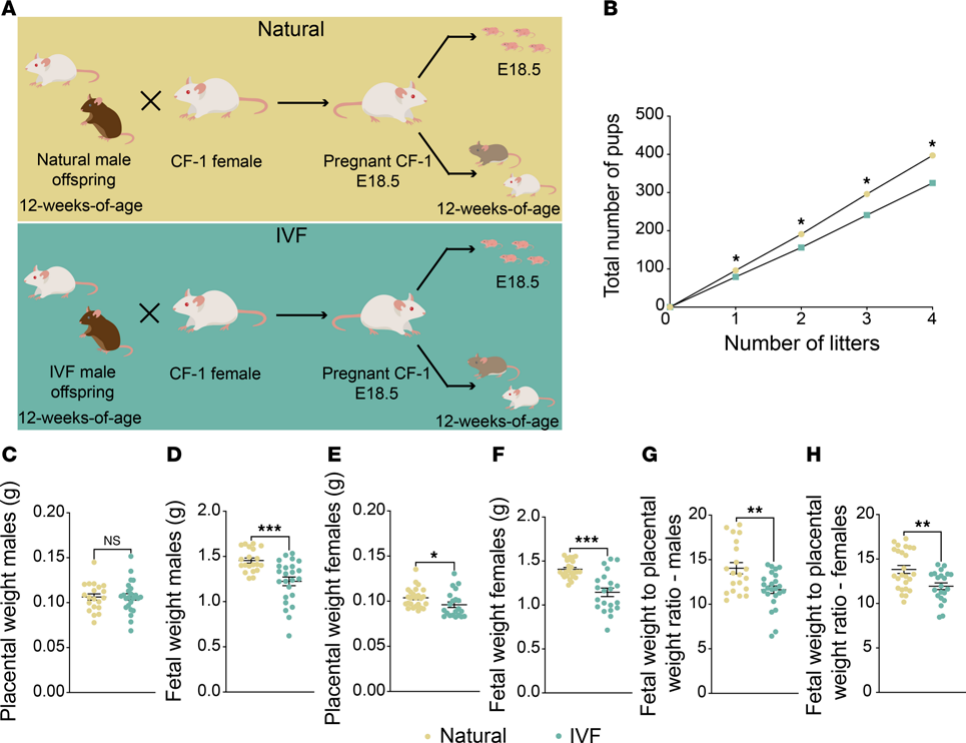
Multigenerational impacts of IVF. (**A**) Mouse model: Top: F2 naturally conceived offspring were generated using natural male offspring and CF-1 female mice, and bottom: F2 IVF offspring were produced using IVF male offspring and CF-1 female mice. E18.5 pregnant females from both groups were C-sectioned, and concepti were delivered and collected for molecular and histological analysis. For adult cohorts naturally delivered pups were maintained until 12 weeks of age. (**B**) Total number of pups is depicted for 4 consecutive litters, with data presented as summary of total pups after each litter from 4 breeding pairs/group. Placental (**C**) and fetal (**D**) weight for male offspring and placental (**E**) and fetal (**F**) weight for female offspring are shown. The fetal weight/placental weight ratio for male (**G**) and female (**H**) offspring. Data are mean ± SEM; *n* = 16–20 per group. Black lines represent the mean of each group. Statistical significance was determined by *t* test; *P < 0.05, **P < 0.01, and ***P < 0.001, when IVF groups are compared with Naturals.

Because sperm DNA methylation was perturbed in IVF-conceived males, we determined whether these changes were transmitted to their offspring. Accordingly, 12-week-old IVF-conceived males and naturals were mated with CF-1 wild-type females, and concepti (F2) were isolated at E18.5 (Figure 5A). We did not observe any differences in the time of conception when comparing pregnancies using IVF males compared with naturals; both had a conception time of 19 days. Litters sired by IVF-conceived males had fewer live pups 12 hours after birth compared with those sired by naturals (Figure 5B). Because we previously identified sex differences in fetal and placental weight in the first generation (7, 36), we performed all analyses by sex. In F2 males, although the mean placental weight was unchanged at E18.5 (Figure 5C), the mean fetal weight was decreased for IVF concepti compared with naturals (Figure 5D). In F2 females, both mean placental weight and mean fetal weight were decreased for IVF concepti compared with naturals at E18.5 (Figure 5, E and F). The reduction in fetal weight, with or without a decrease in placental weight, resulted in significantly reduced fetal/placental weight ratios for F2 IVF compared with naturals (Figure 5, G and H).

**Figure 6:**
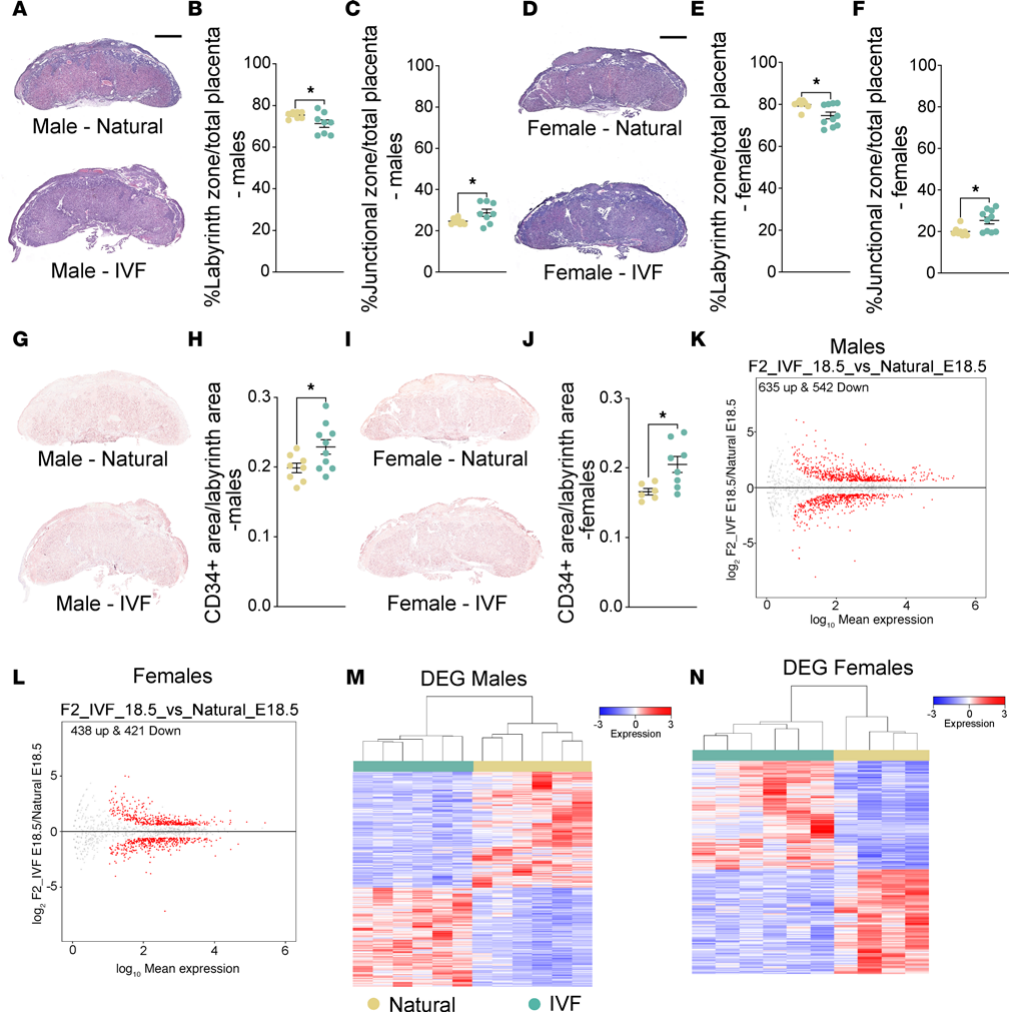
Multigenerational impacts of IVF in placenta. Percentage of placental junctional and labyrinth zone at E18.5 using hematoxylin-eosin staining. Placenta cross sections from (**A**) hematoxylin-eosin–stained tissues (scale bar: 850 μm) from F2 E18.5 male placentas with the percentage of (**B**) labyrinth zone and (**C**) junctional zone shown. (**D**) Hematoxylin-eosin histological sections from F2 E18.5 female placentas with the percentage of (**E**) labyrinth zone and (**F**) junctional zone indicated. Quantitative analysis of labyrinth fetal endothelial cells using CD34 and counterstained with hematoxylin in natural and IVF placentas at E18.5: (**G**) representative images of male placentas from E18.5 natural and IVF and (**H**) quantification of E18.5 fetal endothelial cell positive staining as a percentage of total labyrinth area using ePathology software (5, 24) for male placentas. (**I**) Representative images of female placentas from F2 E18.5 natural and IVF and (**J**) quantification of E18.5 fetal endothelial cell positive staining in females. Original magnification is 4× (**G** and **I**). RNA-Seq using F2 male and female E18.5 placentas: volcano plots showing DEGs for (**K**) F2 male E18.5 placentas and (**L**) F2 female E18.5 placentas IVF versus natural. Heatmap for log_2_-transformed expression levels obtained from RNA-Seq of DEGs in (**M**) F2 male E18.5 placentas and (**N**) F2 female E18.5 placentas. For histology, each data point represents an individual placenta from a minimum of 4 different litters. The black line represents the mean of each group (*n* = 8/group/sex). Statistical significance was determined by *t* test; *P < 0.05 when compared groups against natural.

To address if IVF has a multigenerational impact on the area of the junctional and labyrinth regions in the placenta, as previously observed in IVF offspring (24, 25), we first used hematoxylin-eosin staining at E18.5 (Figure 6, A–D) to assess changes in the ratio of junctional and labyrinth zone areas compared with total placental area. The percentage of labyrinth and junctional zone area was significantly lower and higher, respectively, in F2 IVF placentas compared with naturals (Figure 6, B–F) in both female and male offspring as previously observed for IVF offspring (7). To determine whether blood vessel density was impacted, we performed immunohistochemistry for CD34, a marker of fetal endothelial cells. We have previously shown that CD34 is affected during the early stages of IVF pregnancies (7, 24). We observed that the microvessel density per area was increased in placentas from both IVF F2 males (Figure 6, G and H) and IVF F2 females (Figure 6, I and J) compared with naturals, which impacted placental efficiency.

Finally, given our previous findings of altered gene expression in F1 placentas at E18.5 (7), we determined whether gene expression in F2 placentas from IVF offspring was similarly affected. In F2 males, PCA revealed a clear separation between the IVF and natural groups (Supplemental Figure 6A), with 635 genes upregulated and 542 genes downregulated in the IVF group compared with the natural group (Figure 6, K and M, and Supplemental Table 8). Gene ontology analysis indicated dysregulation of pathways involved in tissue remodeling, nutrient transport, and angiogenesis (Supplemental Figure 6, B and C). Similarly, in F2 females, PCA showed a distinct separation between the IVF and natural groups (Supplemental Figure 6D), with 438 upregulated and 421 downregulated genes observed in the IVF group compared with the natural group (Figure 6, L and N, and Supplemental Table 9). Gene ontology analysis highlighted dysregulation of pathways related to metabolism, transport, and inflammation (Supplemental Figure 6, E and F). Interestingly, in both female and male placentas, vascular endothelial growth factor receptor 1 (*Flt1*) was upregulated, which could contribute to changes in vessel formation.

When comparing these data with previous published data of E18.5 RNA-Seq, we observed that for males approximately 60 DEGs were shared whereas in F2 placentas there was a higher number of new DEGs that could reflect paternal stress contribution as well as epigenetic alterations in the sperm (Supplemental Figure 6G). For females, we observed that when compared with F1 females they had more than 100 shared DEGs but more than 700 unique ones. This difference could reflect the paternal contribution to F2 placentas whereas the F1 placental dysregulation occurs directly because of IVF procedures (Supplemental Figure 6H). Taken together, these findings demonstrate that the alterations observed in male IVF offspring have a multigenerational and persistent negative impact on placental morphology and gene expression in F2 offspring.

### Effect of IVF on male F2 adult offspring metabolism.

**Figure 7:**
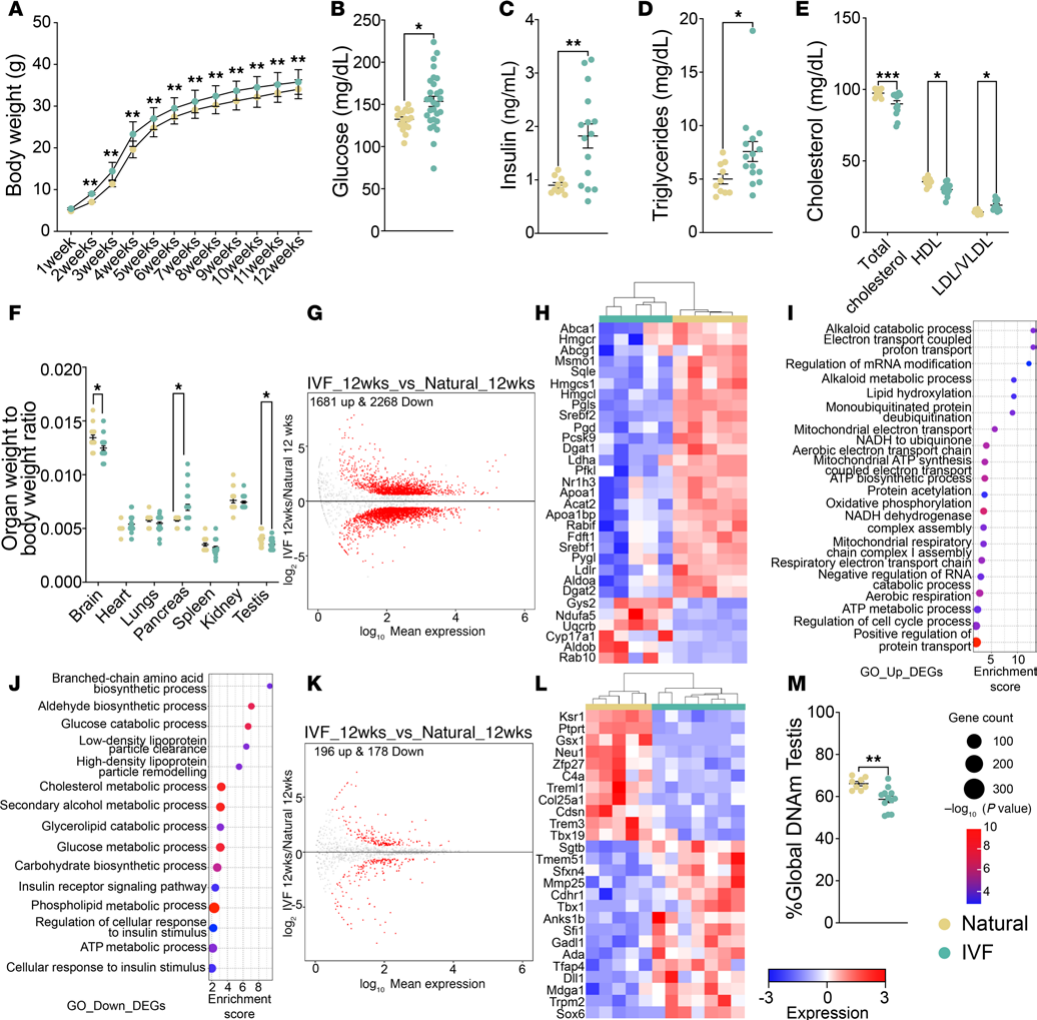
Multigenerational impact of IVF on male offspring. (**A**) Body weights were taken from 1 to 12 weeks of age. Metabolic screening included (**B**) glucose, (**C**) insulin, (**D**) triglycerides, and (**E**) total cholesterol, HDL, and LDL/VLDL. (**F**) The organ weight/body weight ratio is shown. (**G**) Volcano plot showing DEGs for liver RNA-Seq. (**H**) Heatmap for log_2_-transformed expression levels obtained from RNA-Seq of DEGs involved in cholesterol, triglyceride, insulin, and glucose metabolic pathways. Gene ontology analysis for pathways involved in metabolism using (**I**) upregulated DEGs and (**J**) downregulated DEGs. (**K**) Volcano plot showing DEGs for testis RNA-Seq. (**L**) Heatmap for log_2_-transformed expression levels obtained from RNA-Seq of DEGs involved in spermatogenesis and testis differentiation. (**M**) Global DNA methylation of F2 testis at 12 weeks by LUMA. Data are shown as mean ± SEM; *n* = 10–15 per group. The black line represents the mean of each group. Statistical significance was determined by *t* test; *P < 0.05 and **P < 0.01, IVF compared with natural. For RNA-Seq, color boxes at the top denote the experimental group.

We and others have previously determined that metabolism is perturbed in IVF-conceived offspring, with previously published F1 metabolic data (7, 11, 13, 18, 19). To determine whether the F2 progeny sired by IVF-conceived males likewise exhibit adverse health outcomes, we measured the body weight of a cohort of F2 male offspring until 12 weeks of age (Figure 7A) and subsequently euthanized the mice for metabolic analyses. Male F2 offspring sired by IVF-conceived males had higher body weight compared with F2 offspring sired by naturals (Figure 7A). Serum glucose, insulin, total triglyceride, and LDL/VLDL-C were also elevated in F2 offspring relative to naturals (Figure 7, B–E). Total cholesterol was decreased, which was driven by reduced HDL (Figure 7E). We also measured organ weights and calculated their ratio to body weight. F2 offspring sired by IVF-conceived males exhibited decreased brain and testis weight relative to body weight compared with F2 offspring sired by naturals (Figure 7F), whereas pancreas and kidney weights relative to body weight were increased (Figure 7F). Similar phenotypes were also observed in the original IVF male offspring (7, 11).

We previously showed that 12-week IVF offspring exhibited changes in liver gene expression (11). We performed RNA-Seq on a subset of liver and testis samples from male F2 progeny. For liver, PCA showed a clear separation between the IVF and natural groups (Supplemental Figure 7A), with 1,486 upregulated and 1,936 downregulated genes observed when comparing IVF and natural F2 offspring (Figure 7G and Supplemental Table 10). A subset of DEGs associated with metabolic pathways is shown in a heatmap (Figure 7H). Gene ontology analysis revealed dysregulation of pathways involved in cholesterol and phospholipid metabolism, glucose, and insulin processes (Figure 7, I and J). These results are consistent with the metabolic panel and demonstrated that the changes in metabolic measurements are the result of changes in the liver transcriptome. Additionally, we found that some liver DEGs, including *Irs1*, *Irs2*, *Ripk2*, *Rspo1*, and DNA JC genes, have been previously associated with insulin and glucose resistance (38), which would explain the higher insulin and glucose levels revealed in the metabolic panel. Additionally, we tested if there was a normal correlation between glucose and insulin. Although naturals showed a positive correlation, IVF offspring showed a more variable trend and were not correlated as expected with metabolic changes (Supplemental Figure 10A).

To determine whether DEGs observed in F1 and F2 livers were similar, we conducted an overlap analysis using our previously obtained data (11). F2 offspring had more DEGs compared with F1 (3,949 vs. 17), with no overlapping genes. Although the difference in gene expression could reflect the biology of the IVF males, we doubled the number of samples analyzed in this study, which provided more power to detect differences between the 2 groups. Nevertheless, considering the pathway analysis previously performed (11), cholesterol and triglyceride pathways are equally affected in both F1 and F2 offspring.

For testis, PCA showed a clear separation between F2 offspring from IVF and natural groups (Supplemental Figure 6B), and volcano plots comparing F2 offspring revealed 196 upregulated and 178 downregulated genes (Figure 7K and Supplemental Table 11). Some of the most affected genes involved in testicular function and development are shown in a heatmap with differential clustering between groups (Figure 7L). Gene ontology failed to detect a significant association with specific biological functions.

To determine whether the changes in testis DNA methylation persisted into the next generation, we analyzed global DNA methylation in the testis of F2 offspring. Like the male IVF offspring, total global testicular DNA methylation was decreased in F2 IVF offspring compared with the natural conception group (Figure 7M). Taken together, these findings demonstrate that the changes observed in male IVF offspring have a multigenerational and persistent negative impact on metabolism, DNA methylation, and gene expression in the liver and gonads of F2 male offspring.

### Effect of IVF on female F2 adult offspring metabolism.

**Figure 8:**
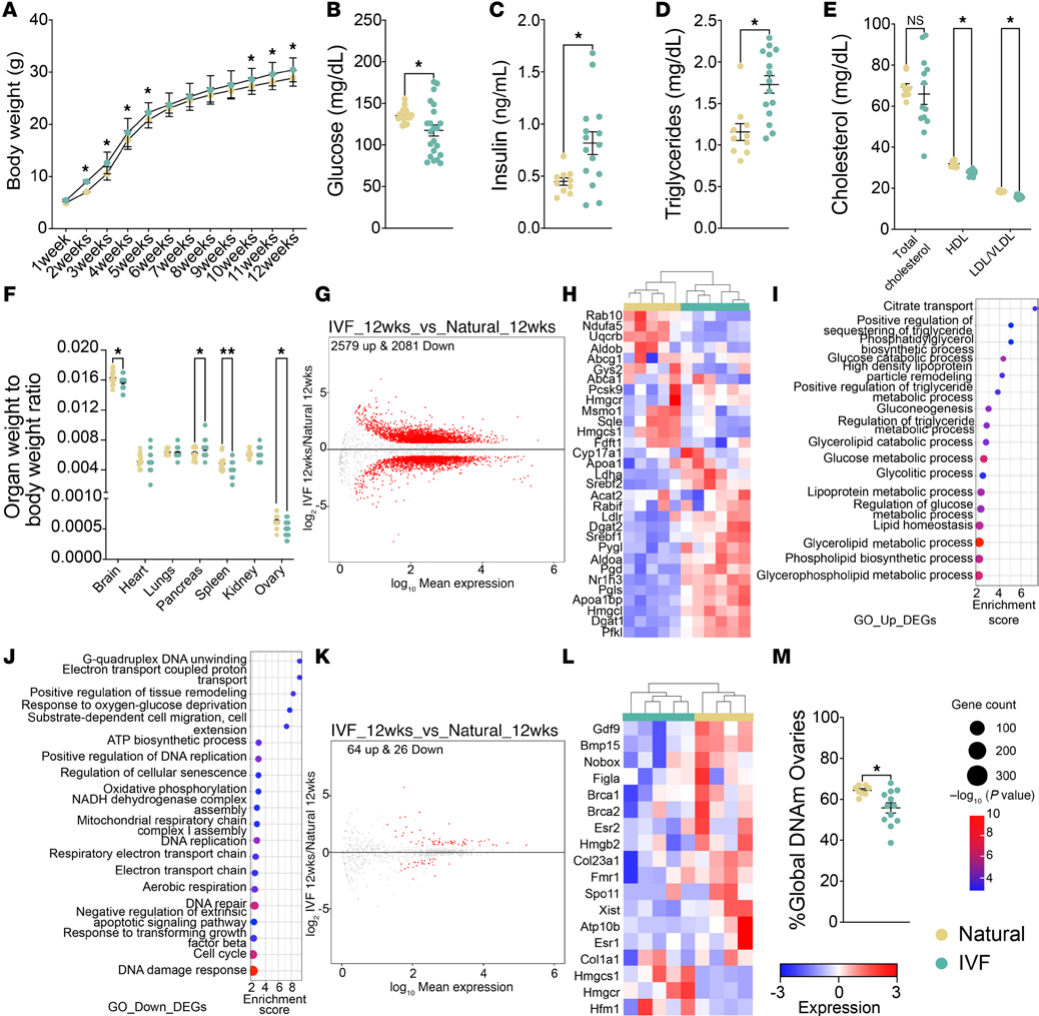
Multigenerational impact of IVF on female offspring. (**A**) Body weights were taken from 1 to 12 weeks of age. Metabolic screening included (**B**) glucose, (**C**) insulin, (**D**) triglycerides, and (**E**) total cholesterol, HDL, and LDL/VLDL. (**F**) The organ weight/body weight ratio is shown. (**G**) Volcano plot showing DEGs for liver RNA-Seq. (**H**) Heatmap for log_2_-transformed expression levels obtained from RNA-Seq of DEGs involved in cholesterol, triglyceride, insulin, and glucose metabolic pathways. Gene ontology analysis for pathways involved in metabolism using (**I**) upregulated DEGs and (**J**) downregulated DEGs. (**K**) Volcano plot showing DEGs for ovary RNA-Seq. (**L**) Heatmap for log_2_-transformed expression levels obtained from RNA-Seq of DEGs involved in ovarian function. (**M**) Global DNA methylation of F2 ovary at 12 weeks by LUMA. Data are depicted as mean ± SEM; *n* = 10–15 per group. The black line represents the mean of each group. Statistical significance was determined by *t* test; *P < 0.05, and **P < 0.01 IVF compared with natural. For RNA-Seq color boxes at the top denote the experimental group.

Given the sexually dimorphic IVF mouse outcomes (7, 11, 18), we conducted a similar set of experiments to those described above for female offspring of IVF-conceived males. These mice had a higher body weight compared with offspring sired by naturals (Figure 8A). Serum glucose was decreased, whereas insulin and total triglycerides were increased and displayed a greater variability in the F2 IVF females compared with naturals (Figure 8, B–D). In contrast, cholesterol, although not statistically different, displayed greater variability, and HDL and LDL/VLDL were decreased in F2 IVF offspring compared with naturals (Figure 8E). Further, F2 offspring sired by IVF-conceived males showed a decrease in ovarian weight relative to body weight compared with naturals (Figure 8F), and as in males, pancreas and kidney weights relative to body weight were increased (Figure 8F). Most of these changes were previously observed in female IVF offspring (7, 11). As in males, we tested if there was a normal correlation between glucose and insulin (Supplemental Figure 10B). No correlation was observed, showing that both glucose and insulin are being dysregulated.

We also performed RNA-Seq on a subset of liver and ovary samples to determine whether gene expression was perturbed in female F2 progeny sired by IVF-conceived males. Similar to male offspring, PCA showed a clear separation between the IVF and natural groups (Supplemental Figure 8A), and volcano plots revealed 2,579 upregulated and 2,081 downregulated genes (Figure 8G and Supplemental Table 12). A heatmap depicts DEGs involved in cholesterol, triglyceride, insulin, and glucose metabolism (Figure 8H). Gene ontology analysis for upregulated (Figure 8I) and downregulated DEGs (Figure 8J) revealed that the most affected pathways are involved in glucose, cholesterol, lipid, and energy pathways, correlating to the observed changes in the metabolic panel. As in males, these results were consistent with the metabolic panel and demonstrated that the changes in metabolic measurements are the result of changes in the liver transcriptome. Although DEGs in males were associated with insulin resistance, DEGs in females were associated with diabetic syndrome.

When RNA-Seq data from ovary were analyzed, PCA again showed a clear separation between F2 offspring sired by IVF-conceived males and those conceived by natural males (Supplemental Figure 8B), with volcano plots showing 64 upregulated and 26 downregulated genes (Figure 8K and Supplemental Table 13). As with liver, we selected a subset of DEGs involved in ovarian function and displayed them on a heatmap (Figure 8L). Gene ontology analysis revealed that ovarian metabolism and hormone production were affected (Supplemental Figure 8C). As in males, we determined if DEGs observed in F1 and F2 offspring livers were similar. Again, F2 showed a higher number of DEGs compared with F1 (4,660 vs. 12), and none of the genes overlapped. Pathway analysis performed in our previous work (11) showed that cholesterol and triglyceride pathways are affected in F1, and the same pathways were affected in our F2 offspring.

To determine whether the DEGs observed in both the liver and gonads were similar between females and males, we conducted an overlap analysis. We found that 2,843 DEGs were shared in the liver, mostly involved in glucose, cholesterol, and triglyceride pathways, consistent with the metabolism panel. Additionally, we identified 1,817 DEGs unique to females and 1,106 unique to males (Supplemental Figure 9A). In gonads, 2 DEGs were shared, with 88 unique to females and 374 to males (Supplemental Figure 9B). Finally, as in males, we assessed whether changes in male IVF offspring could impact global DNA methylation in F2 female gonads. Global DNA methylation in the ovary was decreased in F2 IVF offspring compared with naturals (Figure 8M). These ovarian changes suggest a multigenerational effect.

## Discussion

### IVF negatively affects testicular and sperm parameters compared with naturals.

IVF is often considered an environmental exposure due to the in vitro manipulation of gametes and preimplantation embryos, in which dramatic epigenetic reprogramming occurs (39). A likely outcome of altered epigenetic reprogramming during these early stages of development is that multiple tissues would be affected, e.g., fetus, placenta, and liver (7, 11, 13, 18, 19). The results reported here significantly extend the number of affected tissues by demonstrating that IVF negatively affects male reproductive health and that the reproductive system is extremely sensitive to environmental exposures, with potential consequences for both reproductive and multigenerational health (40).

Male offspring conceived via IVF exhibit significantly decreased levels of testosterone and AR. Testosterone is a male hormone essential for maintaining reproductive health and overall physiological functions (26, 27). Previous studies in models of obesity and metabolic syndrome have shown that males with reduced testosterone levels also exhibit a decline in AR expression because of a positive feedback loop (41). Although these hormonal changes may correlate with previously characterized metabolic syndromes, it is possible that changes during preimplantation and postimplantation development directly influence gene expression pathways involved in testosterone production and AR regulation. Alterations in both the number and functional capacity of the cells responsible for testosterone production could also be a contributing factor in reduced AR expression.

Because of differences in testes’ weight and testosterone levels, we interrogated the seminiferous tubules of IVF male offspring, identifying alterations in the seminiferous epithelium. Studies in mice, rats, and humans have shown that alterations in the seminiferous tubules can impair spermatogenesis, affecting key processes such as spermatid elongation, axoneme formation, and interactions between Sertoli cells and germ cells (28). Depending on the severity of the disruption, these changes can also impact the timing and overall efficiency of spermatogenesis (28). Most environmental exposures and metabolic syndromes are associated with an increase in tubule area; however, in our study, we observe not only an increase in overall tubule area but also an expansion of the epithelial membrane that may reflect dysregulated spermatogenesis and cell division.

Another potential cause of the increase in both testicular weight and overall tubule area is the overdeposition of collagen and laminin, which is typically seen in fibrosis of the testis (42). At 39 weeks, we observe higher collagen deposition in the seminiferous epithelium membrane, which could disrupt normal cell distribution and lead to the tubule overgrowth observed in IVF offspring. Excessive collagen deposition is linked to various pathologies, including fibrosis, Klinefelter syndrome, inflammation, infection, aging, and physical stress (42, 43). Our results are consistent with fibrosis and early aging. Future studies will address whether this phenotype is associated with specific gene mutations or changes in gene expression related to collagen regulation and the maintenance of the epithelial membrane.

Changes in testosterone levels and morphology provide a plausible explanation for the negative impact on spermatogenesis. Consistently, RNA-Seq on testicular tissues identified dysregulation of genes involved in hormone production and regulation, as well as extracellular matrix composition and maintenance. Among the dysregulated genes in testis, several were related to collagen production, reflecting the reported phenotype. Although the collagen and morphology changes in our model are explained by the dysregulation of genes important for these pathways, we also hypothesized that some of these changes could represent a response to the metabolic syndrome affecting the IVF male offspring (44). Previous studies in models of obesity and alcohol exposure have demonstrated that progeny of exposed animals exhibit gene expression dysregulation across multiple systems, including the testis. These changes have been associated with inherited phenotypes driven by altered sperm maturation because of disruptions in overall spermatogenesis (44–47).

Although the impact of IVF on sperm in IVF-conceived males could be due to abnormal seminiferous tubules, metabolic syndromes and environmental exposures have an impact on the number, morphology, and molecular integrity of the sperm (48, 49). In humans, IVF male offspring did not present with changes in sperm count but did have an increase in sperm abnormalities (23). Although we also saw no differences in sperm count at 12 weeks, we observed altered morphology, with most of the changes related to head and acrosome abnormalities. Ultimately, an increase in the number of sperm abnormalities will impact the success of fertilization and compromise male fertility (50).

Because of previously identified changes in DNA methylation in IVF offspring reported by our group and others (7, 13, 24, 25), we examined DNA methylation in both testis and sperm. Both testicular and sperm DNA methylation were globally impacted, likely resulting from alterations in normal reprogramming during embryo formation. We also examined the sperm DNA methylation landscape in greater depth to determine the possibility of transmitting an abnormal epigenome to the next generation. A normal epigenetic landscape in sperm includes DNA methylation marks, retained histones and protamines, and noncoding RNAs (51). At both 12 and 39 weeks, we observed changes in DNA methylation, with a greater number of affected regions at the later age. Because environmental exposures can alter the DNA methylation patterns of sperm, negatively impacting the next generation (51, 52), IVF may induce similar effects, compromising sperm integrity and contributing to the multigenerational changes reported here. Interestingly, no specific genomic feature was affected; instead, all genomic regions were affected as we previously reported for other tissues (5, 7).

### IVF has a multigenerational negative impact.

To our knowledge, few studies have focused on the F2 generation of IVF offspring. Previous studies in mice reported that offspring from IVF/ICSI-conceived males exhibited altered fasting glucose and insulin levels (17, 21), with more pronounced changes when the offspring were exposed to a high-calorie diet (17, 21, 53). Because male gametes from IVF-conceived males have defects, e.g., changes in DNA methylation, we assessed whether such changes are observed in F2 offspring from IVF sires. At term (E18.5), sired offspring exhibited lower fetal and placental weights, which affected the fetal-to-placental weight ratio. Changes in this ratio are associated with altered placental efficiency (54) and are consistent with the small for gestational age (SGA) classification; SGA is linked to multiple paternal factors, including age, obesity, and other metabolic syndromes (55). At E18.5, we observe changes in gene expression in the placentas of F2 offspring from IVF compared with those from natural conception. These changes likely disrupt normal placental function and efficiency, potentially impeding the nutrient supply to the fetus and predisposing the offspring to metabolic dysregulation. Furthermore, abnormal paternal DNA methylation observed in the sperm may influence placental development and function, as reflected in the observed phenotype.

We hypothesize that the metabolic changes observed in the F2 generation result from metabolic alterations induced by IVF in the first generation, which are transmitted via affected sperm. Because sperm carry both genetic information and epigenetic marks, such as DNA methylation and histone modifications, abnormal DNA methylation in the father can be transmitted to the zygote, leading to developmental dysregulation, particularly in metabolism and growth. Additionally, we propose that these changes are exacerbated by paternal metabolic syndrome, which could increase systemic inflammation, oxidative stress, or hormonal imbalances, further altering the epigenetic landscape of the sperm and contributing to the observed metabolic and developmental phenotypes in F2 offspring.

Finally, we followed F2 mice to 12 weeks of age — a time at which we have previously identified significant changes in metabolic phenotypes in both female and male IVF offspring (7, 11). We observed that whereas most individuals appeared healthy, some exhibited altered metabolism, which mirrored the changes observed in the F1 generation (7, 11). These metabolic changes reflect those previously associated with metabolic syndrome in both humans and mice (13, 18, 56). Interestingly, we observed sex-specific adverse outcomes in the F2 from IVF offspring, including a higher risk of insulin and glucose resistance in males and a diabetic phenotype in females. These sex-specific differences can arise by sex-linked genes or hormones. We also observed more severe metabolic and gene expression changes in the F2 generation, as evidenced by the F2 liver RNA-Seq data. These effects could result from the cumulative impact of IVF in the F1 generation, together with a potential contribution of metabolic syndrome in the males, which may be inherited through the germline. These findings underscore that the negative effects of IVF not only persist but also may intensify in subsequent generations.

The changes observed in our model are likely the result of the cumulative effects of ART, such as longer culture to reach blastocyst stage and the metabolic syndromes developed by the IVF offspring fathers. Currently, no human studies have focused on the multigenerational effects of IVF largely because of the age of this population. The mouse model is advantageous for these studies because mice are sexually mature at 6–8 weeks, samples are easily accessible, external factors can be controlled, and the specific effects of ART interventions can be identified. Our findings highlight the importance of monitoring the long-term health of IVF offspring and subsequent generations.

**Figure 9:**
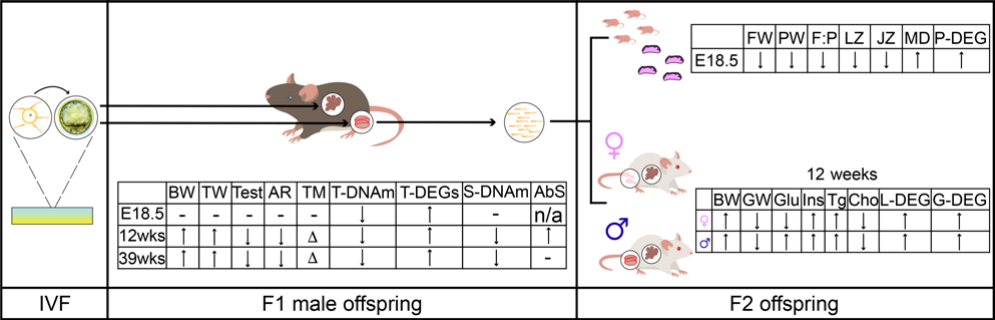
Summary of major findings. The use of IVF induced changes in testicular and sperm morphology and molecular profiles. Arrows indicate the direction of the changes compared with Naturals, with more arrows showing more significant changes. F1 male offspring: BW, body weight; TW, testicular weight; Test, testosterone; AR, androgen receptor protein levels; TM, testicular morphology; T-DNAm, testicular DNA methylation; T-DEGs, testicular differentially expressed genes; S-DNAm, sperm DNA methylation; AbS, abnormal sperm morphology. F2 offspring, E18.5 FW, fetal weight; PW, placental weight; F:P, fetal weight/placental weight ratio; LZ, %labyrinth zone; JZ, %junctional zone; MD, microvessel density; P-DEG, placental differentially expressed genes. 12 weeks: BW, body weight; GW, gonad (testis or ovary) weight; Glu, glucose; Ins, insulin; Tg, triglycerides; Cho, cholesterol; L-DEGs, liver differentially expressed genes; G-DEGs, gonadal (testis or ovary) differentially expressed genes; ↑, significantly higher; ↓, significantly lower; Δ, changed; –, not measured in this project; n/a, not applicable.

In summary (Figure 9), we demonstrate that IVF offspring exhibit molecular and morphological changes in testis and sperm that affect not only the reproductive system but also other systemic functions. Additionally, these changes have a significant impact on the progeny of IVF offspring, leading to an increased risk of developing metabolic syndromes in adulthood. This work underscores the necessity of using a suitable animal model to evaluate the potential risks associated with IVF and other ART in the reproductive systems of humans and mice. It also emphasizes the broad effects of IVF on various systems that have been overlooked and the importance of improving ART technologies to ensure the health of both offspring and future generations.

## Methods

### Sex as a biological variable.

For this study, sex was considered as a biological variable in our experimental design because of sexually dimorphic adverse outcomes previously observed (7, 11, 18). We included only males to ensure a comprehensive evaluation of the effects of IVF on the next generation. Our results cannot be generalized, as reproductive development in males and females is different. Nevertheless, evaluation of the multigenerational impact of IVF included both female and male offspring, as the second generation from an IVF male has not been previously studied, and no sex-specific effects have been identified.

### Animals.

Breeding stocks of CF-1 female and male (Charles River Laboratories), C57BL/6-Tg(CAG-EGFP)131Osb/LeySopJ male and SJL female (The Jackson Laboratory) to generate SJL/B6 males as sperm donors, and CD-1 vasectomized male mice (Charles River Laboratories) were maintained in a pathogen-free facility. All animals were housed in polysulfone cages and had access to drinking water and chow (Laboratory Autoclavable Rodent Diet 5010, LabDiet) ad libitum.

### Generation of natural offspring.

Offspring conceived naturally (naturals) were generated by mating CF-1 females in their natural estrous cycle with 8-week-old SJL/B6 males heterozygous for GFP. Detection of a vaginal plug marked E0.5, and the embryos developed in vivo without embryo transfer (Figure 1A).

### Generation of IVF offspring.

As previously described (7, 11, 24, 25), IVF offspring were generated according to optimized protocols recommended by The Jackson Laboratory (57). CF-1 females were superovulated with 5 IU equine chorionic gonadotropin (eCG) followed by 5 IU human chorionic gonadotropin 46 hours after eCG injection. On the day of IVF, sperm were collected from the vas deferens and caudal epididymis of a male SJL/B6 heterozygous for GFP. The collection was performed using EmbryoMax Human Tubal Fluid media (HTF; EMD Millipore) containing 3% w/v bovine serum albumin (BSA; AlbuMax, Gibco) and under mineral oil (Irvine Scientific). Sperm were then capacitated for at least 1 hour. Eggs were collected and fertilized with capacitated SJL/B6 sperm in HTF medium. After 4 hours, eggs with evident pronuclei (fertilized) were washed in HTF medium and EmbryoMax KSOM medium containing half amino acids (KSOM+AA, EMD Millipore) before culturing to the blastocyst stage in KSOM+AA covered with mineral oil at 37°C in an atmosphere of 5% CO_2_, 5% O_2_, and 90% N_2_. After 3.5 days of culture, blastocysts were removed from the KSOM+AA culture droplet and briefly washed in Multipurpose Handling Medium Complete (Irvine Scientific) with gentamicin prior to embryo transfer. Pseudopregnant recipients (3.5 days postcopulation) were generated by mating CF-1 females with CD-1 vasectomized males. Each pseudopregnant recipient received 10 blastocysts by nonsurgical embryo transfer (Figure 1B). Day of blastocyst transfer was defined as E3.5.

### Fostering of natural and IVF offspring.

Fostering of natural and IVF offspring was performed as previously described (11). We generated 3 cohorts using individuals from 5 litters for each time point: (a) E18.5 cohort of 12 natural and 10 IVF males, (b) 12 weeks of age cohort of 12 natural and 12 IVF males, and (c) 39 weeks of age cohort of 12 natural and 19 IVF males.

### Tissue collection.

Naturally mated females or pseudopregnant recipients with IVF embryos were euthanized at E18.5, and fetuses and placentas were collected. Fetal and placental wet weights were recorded. Gonadal tissue was snap-frozen in liquid nitrogen. Adults at 12 weeks and 39 weeks of age were euthanized. Testes were collected and seminiferous tubules were collected from one of the testes and snap-frozen in liquid nitrogen. The other testis was fixed in 10% of phosphate-buffered formalin for histological analyses. Sperm was collected from the vas deferens and cauda, then processed using somatic cell lysis buffer, and the isolated sperm was snap-frozen. Blood was collected; serum was then isolated by centrifugation at 4°C and kept at –80°C for hormone measurements.

### Sperm morphology analysis.

Previously collected sperm from 12 weeks of age mice was used to obtain smears for morphological analysis using hematoxylin-eosin staining. We assessed abnormal sperm, categorizing them as follows: abnormal head (%head), abnormal tail (%tail), abnormal midpiece (%midpiece), and total abnormal sperm (%abnormal). Our analysis referenced previous studies by other authors who also assessed sperm morphology (58, 59).

### Hematoxylin-eosin, PR, and MT staining.

Testicular sections were stained with hematoxylin-eosin as described (7, 24). Testicular sections were imaged using an EVOS FL Auto Cell Imaging System and software (Thermo Fisher Scientific) at 4× original magnification and analyzed using FIJI/ImageJ (NIH). Changes in the seminiferous tubules’ area, thickness, and overall structure were recorded. ImageJ was used to identify changes in the intensity of the stain associated with collagen levels.

To assess changes in collagen deposition and potential fibrosis, PR staining was used on testicular sections as previously described (60). Briefly, tissue sections were deparaffinized in xylene and rehydrated through a graded series of ethanol concentrations (100%, 70%, and 30%), followed by rinsing with deionized water. Slides were then immersed in a PR staining solution, prepared by dissolving Sirius Red F3B (Direct Red 80, C.I. 35782, MilliporeSigma) in a 1.3% saturated aqueous picric acid solution (MilliporeSigma) at 0.1% w/v. After a 30-minute incubation in the staining solution at room temperature, the slides were destained with 0.05N HCl. The tissue sections were then dehydrated in 100% ethanol, cleared in xylene, and mounted with Permount mounting medium. Stained slides were imaged using an EVOS FL Auto Cell Imaging System (Thermo Fisher Scientific). The percentage of PR-positive signal was calculated using 3 testicular sections per animal with ImageJ.

To visualize collagen and validate PR staining, the MT assay was performed according to the manufacturer’s instructions (Polysciences, Inc.) on 3 testicular sections from each male. Slides were stained by the Pathology Core at the Children’s Hospital of Philadelphia (CHOP). Briefly, testicular sections were deparaffinized in xylene and rehydrated in graded ethanol (100% and 95%), followed by mordanting in preheated Bouin’s solution for 15 minutes at 60°C. Slides were then gently washed under running tap water for 5 minutes and stained with Weigert’s Iron Hematoxylin for 10 minutes. After several washes with tap water, the slides were stained in Biebrich Scarlet-Acid Fuchsin solution (MilliporeSigma) for 5 minutes and rinsed in distilled water. Samples were then incubated with phosphotungstic/phosphomolybdic acid for 10 minutes and finally stained with Aniline Blue for 5 minutes. After several rinses with distilled water, the slides were transferred to 1% acetic acid for 1 minute, dehydrated in ethanol, cleared in xylene, and mounted in VECTASHIELD mounting medium (Vector Laboratories). Slides were imaged in bright-field using an EVOS FL Auto Cell Imaging System. Images were analyzed using FIJI/ImageJ and the plugin colour_deconvulation2 (61, 62). The percentage of MT-positive signal was calculated using 3 testicular sections per animal.

### Testis immunohistochemistry.

For testicular immunochemistry, histological sections were rehydrated using xylene, followed by graded series of ethanol and then with PBS. Antigen retrieval was performed using Antigen Unmasking Solution (Vector Laboratories). Sections were subsequently washed, quenched with a 30% hydrogen peroxide/methanol solution, washed again in PBS, and blocked using 15% normal goat serum in 0.3% Triton-PBS for 1 hour at room temperature. The primary antibody, anti-SYCP3 (Abcam, ab97672) was applied at a dilution of 1:250 for 1 hour at room temperature, followed by overnight incubation at 4°C. Negative control slides received 15% normal goat serum without primary antibody. The following day, slides were washed with PBS, and secondary antibodies were applied: Goat anti-Mouse IgG (H+L) Highly Cross-Adsorbed Secondary Antibody, Alexa Fluor Plus 647 (Thermo Fisher Scientific) at 1:250, along with Lectin PNA From *Arachis hypogaea* (peanut) Alexa Fluor 488 Conjugate (L21409, Thermo Fisher Scientific) at 1:250 in blocking solution, for 1 hour. After washing with PBS, slides were incubated with DAPI at 1:100 in blocking solution for 30 minutes. Finally, slides were washed in PBS and distilled water, then mounted using ProLong Diamond Antifade Mountant (Thermo Fisher Scientific). Robust positive staining was observed in spermatogonia, spermatocytes, and round spermatids, consistent with previous descriptions (63, 64). SYCP3-positive cells (spermatogonia and spermatocytes) and the total number of round spermatids (PNA-positive cells with a distinct DAPI chromocenter) in stage II–III and IV–VIII seminiferous tubules (>10 sections per stage, *n* = 6 per group) were scored by 2 people.

### Placenta histology and immunohistochemistry.

Placenta histology was performed as previously described (7, 24). At E18.5, placentas were collected, with half snap-frozen for molecular analysis and the other half fixed in 10% formalin. The fixed placentas were dehydrated through a graded series of ethanol concentrations, cleared with xylene, and embedded in paraffin. Serial histological sections of 5 μm were prepared using a microtome. Placentas were stained with hematoxylin-eosin and imaged using an EVOS FL Auto Cell Imaging System. Images were analyzed using FIJI/ImageJ to measure the total placental, labyrinth zone, and junctional zone area. Each data point represents the average of 3 placental sections, spaced 50 μm apart. The ratios of the junctional zone and labyrinth zone to the total placental area were calculated and expressed as percentages.

Placental sections were immunostained to detect vascular changes using the endothelial cell–specific marker CD34 as previously described (7, 24). Briefly, placental sections were rehydrated through a graded series of xylenes, graded ethanol series, and PBS. Antigen retrieval was performed using an Antigen Unmasking Solution (Vector Laboratories). Sections were washed and quenched with a 30% hydrogen peroxide/methanol solution, then washed in PBS twice, and blocked using 15% normal goat serum for 1 hour at room temperature. Anti-CD34 antibody (Abcam, ab81289) was applied to sections at a dilution of 1:200 for 1 hour at room temperature, then overnight at 4°C. For negative control slides, 15% normal goat serum was reapplied without the CD34 primary antibody. The next day, slides were washed with PBS, secondary goat anti-Rabbit IgG antibody (H+L) biotinylated (Vector Laboratories) was applied to all slides at 1:1,000 for 1 hour, and the ready-to-use VECTASTAIN Universal ABC kit and Vector NovaRED Peroxidase (HRP) Substrate Kits were used according to the manufacturer’s instructions (Vector Laboratories). Robust positive staining was observed in fetal endothelial cells of the labyrinth, as previously reported (24). Slides were counterstained with Harris Hematoxylin (MilliporeSigma), dehydrated through a graded ethanol series and xylene, and mounted using Fisher Chemical Permount Mounting Medium (Thermo Fisher Scientific). Placental sections were scanned and digitized using the Aperio Scanscope CS-O slide scanner (Leica) at the CHOP Pathology Core Laboratory. The labyrinth area was outlined using Aperio Imagescope software (Leica), and the Aperio Blood Vessel Analysis algorithm was applied as previously described (7, 24). Data are presented as the algorithm-measured areas of CD34-positive staining relative to the labyrinth area. Each data point represents the average of 2 placental sections, spaced 100 μm apart. The samples assessed at E18.5 included natural (*n* = 12; 6 males and 6 females) and IVF (*n* = 12; 6 males and 6 females).

### Testosterone levels.

Serum collected from blood on the day of euthanasia was used to measure changes in testosterone levels using the commercially available Mouse/Rat Testosterone ELISA Kit (ab285350, Abcam) and following the manufacturer’s guidelines.

### DNA and RNA isolation from testis, placenta, and sperm.

DNA and RNA were isolated from one-quarter of each snap-frozen testis, as previously described (65). Sperm was collected from the vas deferens and caudal epididymis at 12 and 39 weeks of age and capacitated in EmbryoMax HTF (EMD Millipore) with 3% w/v BSA for 30 minutes at 37°C. Motile sperm was collected by removing the supernatant, spun down for 5 minutes at 650*g*, and incubated for 15 minutes on ice with somatic cell lysis buffer (0.1% SDS, 0.5% Triton X-100) to lyse and remove somatic cells. After treatment with the lysis buffer, sperm was counted, spun down for 5 minutes at 10,000*g*, and snap-frozen for storage at –80°C until further processing. For DNA isolation, seminiferous tubules were digested in lysis buffer (50 mM Tris at pH 8.0, 100 mM EDTA, 0.5% SDS) with proteinase K (180 U/mL; MilliporeSigma) overnight at 55°C. Sperm pellets were resuspended in sperm lysis buffer (20 mM Tris-HCl, pH 8.0, 200 mM NaCl, 20 mM EDTA, 4% SDS) with 5 μL of β-mercaptoethanol and proteinase K (180 U/mL) and incubated overnight at 55°C. Genomic DNA was isolated using phenol/chloroform/alcohol (25:24:1; MilliporeSigma), followed by ethanol precipitation and resuspension in buffer (10 mM Tris-HCl, pH 8.0, 0.5 mM EDTA).

RNA was isolated from a cross section of the testis or placenta using an adapted protocol of TRIzol (Thermo Fisher Scientific) and miRNeasy Kit (QIAGEN), following the manufacturer’s protocol. DNase treatment was performed during RNA isolation to eliminate genomic DNA contamination. RNA quality and concentration were determined by RNA ScreenTape analysis using a TapeStation (Agilent Technologies).

### LUMA.

Genomic DNA (1 μg) from testis, sperm, and ovaries (second generation) was used to measure global DNA methylation by LUMA as previously described (7, 24, 25).

### Genome-wide DNA methylation profiling and analysis.

We performed genome-wide DNA methylation profiling on a random subset of 12-week and 39-week-old offspring sperm (*n* = 6 males for each group, 12 for each age), as previously described (7, 35). The samples were processed at the Children’s Hospital of Philadelphia (CHOP) Center for Applied Genomics Genotyping Core. Bisulfite-treated DNA (1 μg) was pipetted onto an Illumina Infinium Mouse Methylation-12v1-0 BeadChip that was run on an Illumina iScan System using the manufacturer’s standard protocol as previously described (5, 35, 36).

Raw IDAT files were processed, as previously described (5, 35, 66). Changes in DNA methylation were identified by comparing the methylation levels probe by probe between naturals and IVF offspring. We analyzed the data using the pipeline recommended by the SeSAMe package (67), as previously described (7, 35). CG identity was assessed using the tool KYCG part of the SeSAMe R package. Pathway analyses were performed using the package for R: clusterProfiler.

### RNA-Seq.

We performed RNA-Seq on a random subset of E18.5, 12-week, and 39-week testis from *n* = 5 individuals for each group, different litters and tissue, a random subset of E18.5 placentas from *n* = 4–6 F2 for each group and 5 litters to determine changes in gene expression, and a random subset of liver, testis, and ovaries from the second generation. As previously described (7, 11), total RNA (4 μg) was used to prepare mRNA-Seq libraries using mRNA-Seq library synthesis kit and Single-Indexed adapter kit (both KAPA Biosystems). Library quality control was conducted using High Sensitivity DNA ScreenTape for TapeStation (Agilent Technologies) and Library Quantification Kit (KAPA Biosystems). Sequencing was performed using the NovaSeq 1000 platform (Illumina).

RNA-Seq reads were analyzed as previously described (7, 11). Numbers for total sequenced, aligned, and counted reads for each sample are listed in Supplemental Table 1.

### RT-qPCR.

Extracted testicular RNA from naturals and IVF offspring at 12 and 39 weeks were cDNA converted as previously described (5, 7, 11). Primers are listed in Supplemental Table 2. Relative expression was calculated using the quantified expression from the endogenous control *Nono* and Actin B (*ActB*) that showed stable expression levels in mouse across multiple samples. RT-qPCR was conducted on all samples, including those used for RNA-Seq: 12 weeks natural (*n* = 12) and IVF (*n* = 12) and 39 weeks naturals (*n* = 12) and IVF (*n* = 12).

### Western blot.

Seminiferous tubules from 12- and 39-week males were used for protein analysis by Western blot as previously described (11). Protein lysate (20 μg) was used per sample, and each membrane was probed with primary antibodies diluted in 5% nonfat dairy milk in TBS-T for GAPDH (1:5,000, Cell Signaling Technology) and AR (1:1,000; Abcam), VCAM1 (1:1,000; Abcam), or COL1A1 (1:1,000; Abcam). Levels of AR, COL1A1, and VCAM1 were determined relative to GAPDH levels and compared between groups.

### Multigenerational studies in natural and IVF offspring.

Natural and IVF offspring at 12 weeks of age were mated with wild-type CF-1 females for 4 consecutive litters. At E18.5 some pregnant females were euthanized, and both fetus and placenta were collected to assess prevalence of the phenotype on the next generation. Other litters were weaned at 21 days of age, and using a calibrated digital scale, body weights were measured at birth and weekly from 3 to 12 weeks of age. Five animals were housed per cage to ensure no differences in food and water consumption as previously described (7, 11). Twelve-week-old animals were euthanized, and blood glucose levels were obtained by tail snip using a hand-held glucometer (ReliOn). Whole blood was collected by cardiac puncture, then centrifuged at 10,000*g*, and serum was collected and stored at −80°C for subsequent assays. Total triglycerides were assayed using enzymatic colorimetric assay kits from Stanbio (Boerne), and insulin was assayed using enzymatic colorimetric assay kits from Crystal Chem. Total cholesterol, HDL, and LDL/VLDL were assayed using the HDL and LDL/VLDL Cholesterol Assay Kit (Abcam ab65390).

Organ weights were recorded, and blood was collected for metabolic and hormonal panels. For molecular analysis, part of the liver, previously homogenized pancreas in TRIzol, one of the ovaries, and one of the testes snap-frozen in liquid nitrogen were used. For morphological and histological analysis, the remaining liver, pancreas, one of the ovaries, and one of the testes were fixed in 10% formaldehyde in PBS.

### Statistics.

All samples were statistically analyzed by 2-tailed *t* test. Probabilities of genomic regions in the array were compared using a Bernoulli distribution using R v 4.2.2 (R foundation for Statistical Computing; www.R-project.org/). Significant differences comparing natural and IVF offspring were denoted as statistically significant if *P* < 0.05. Differences in variability was calculated by *F* test; statistically significant differences comparing natural and IVF were denoted if *P* < 0.05. Sperm correlation analysis was performed using a Pearson *r* test and considered significant if *P* < 0.05. All statistical analyses were performed using GraphPad Prism version 9.1.

### Study approval.

All animal work was conducted with the approval of the Institutional Animal Care and Use Committee (IACUC) at the University of Pennsylvania. IACUC protocol 80354 has been previously revised and approved.

### Data availability.

All values for all data points in graphs are reported in the Supporting Data Values file. Additionally, the data array and sequencing data underlying this article are available at National Center for Biotechnology Information Gene Expression Omnibus at https://www.ncbi.nlm.nih.gov/geo/, under accession number GSE280286.

## Author contributions

EARC and MSB designed research studies. EARC, AJS, CNH, and LR conducted experiments. EARC and AJS acquired data. EARC wrote the manuscript; and EARC, CNH, AJS, LR, RMS, and MSB edited and reviewed the manuscript prior to submission. EARC is listed as first author based on his conceptualization and initiation of the project.

## Supplementary Material

Supplemental data

Unedited blot and gel images

Supplemental table 1

Supplemental table 10

Supplemental table 11

Supplemental table 12

Supplemental table 13

Supplemental table 2

Supplemental table 3

Supplemental table 4

Supplemental table 5

Supplemental table 6

Supplemental table 7

Supplemental table 8

Supplemental table 9

Supporting data values

## Version 1 03/04/2025

In-Press Preview

## Version 2 04/22/2025

Electronic publication

## References

[2] Eskew AM, Jungheim ES (2017). A history of developments to improve in vitro fertilization. Mo Med.

[3] Rhon-Calderon EA (2019). The effects of assisted reproductive technologies on genomic imprinting in the placenta. Placenta.

[4] Gosden R (2003). Rare congenital disorders, imprinted genes, and assisted reproductive technology. Lancet.

[5] Vrooman LA (2022). Placental abnormalities are associated with specific windows of embryo culture in a mouse model. Frontiers in cell and developmental biology. Front Cell Dev Biol.

[6] Chronopoulou E, Harper JC (2015). IVF culture media: past, present and future. Hum Reprod Update.

[7] Rhon-Calderon EA (2023). Trophectoderm biopsy of blastocysts following IVF and embryo culture increases epigenetic dysregulation in a mouse model. Hum Reprod.

[8] Kushnir VA (2022). The future of IVF: the new normal in human reproduction. Reprod Sci.

[9] Kanatsu-Shinohara M (2023). Intracytoplasmic sperm injection induces transgenerational abnormalities in mice. J Clin Invest.

[10] Bryda EC (2013). The Mighty Mouse: the impact of rodents on advances in biomedical research. Mo Med.

[11] Narapareddy L (2021). Sex-specific effects of in vitro fertilization on adult metabolic outcomes and hepatic transcriptome and proteome in mouse. FASEB J.

[12] Harner R (2021). Ovulation induction is associated with altered growth but with preservation of normal metabolic function in murine offspring. F S Sci.

[13] Lira-Albarrán S (2022). DNA methylation profile of liver of mice conceived by in vitro fertilization. J Dev Orig Health Dis.

[14] Cui L (2020). Increased risk of metabolic dysfunction in children conceived by assisted reproductive technology. Diabetologia.

[15] Kianpour M (2023). Metabolic syndrome and assisted reproductive techniques. J Family Reprod Health.

[16] Qin N (2021). Abnormal glucose metabolism in male mice offspring conceived by in vitro fertilization and frozen-thawed embryo transfer. Front Cell Dev Biol.

[17] Heber MF, Ptak GE (2020). The effects of assisted reproduction technologies on metabolic health and disease†. Biol Reprod.

[18] Donjacour A (2014). The ovarian reserve of primordial follicles and the dynamic reserve of antral growing follicles: what is the link?. Biol Reprod.

[19] Aljahdali A (2020). The duration of embryo culture after mouse IVF differentially affects cardiovascular and metabolic health in male offspring. Hum Reprod.

[20] Rubino P (2016). The ICSI procedure from past to future: a systematic review of the more controversial aspects. Hum Reprod Update.

[21] Ban M (2024). IVF exposure induced intergenerational effects on metabolic phenotype in mice. Reprod Biomed Online.

[22] Balli M (2022). Opportunities and limits of conventional IVF versus ICSI: it is time to come off the fence. J Clin Med.

[23] Catford SR (2018). Long-term follow-up of ICSI-conceived offspring compared with spontaneously conceived offspring: a systematic review of health outcomes beyond the neonatal period. Andrology.

[24] Vrooman LA (2020). Assisted reproductive technologies induce temporally specific placental defects and the preeclampsia risk marker sFLT1 in mouse. Development.

[25] de Waal E (2015). The cumulative effect of assisted reproduction procedures on placental development and epigenetic perturbations in a mouse model. Hum Mol Genet.

[27] Ide H (2023). The impact of testosterone in men’s health. Endocr J.

[28] Xie B-G (2014). Pathological changes of testicular tissue in normal adult mice: a retrospective analysis. Exp Ther Med.

[29] Nantel F (1996). Spermiogenesis deficiency and germ-cell apoptosis in CREM-mutant mice. Nature.

[30] Matzkin ME (2021). Hallmarks of testicular aging: the challenge of anti-inflammatory and antioxidant therapies using natural and/or pharmacological compounds to improve the physiopathological status of the aged male gonad. Cells.

[31] Siu MK, Cheng CY (2008). Extracellular matrix and its role in spermatogenesis. Adv Exp Med Biol.

[32] Li H (2020). Bioactive fragments of laminin and collagen chains: lesson from the testis. Reproduction.

[33] López De Padilla CM (2021). Picrosirius red staining: revisiting its application to the qualitative and quantitative assessment of collagen type I and type III in tendon. J Histochem Cytochem.

[34] Van De Vlekkert D (2020). Analysis of generalized fibrosis in mouse tissue sections with Masson’s trichrome staining. Bio Protoc.

[35] Prasasya RD (2024). Iterative oxidation by TET1 is required for reprogramming of imprinting control regions and patterning of mouse sperm hypomethylated regions. Dev Cell.

[36] Mani S (2022). Embryo cryopreservation leads to sex-specific DNA methylation perturbations in both human and mouse placentas. Hum Mol Genet.

[37] MacPhillamy C (2024). DNA methylation analysis to differentiate reference, breed, and parent-of-origin effects in the bovine pangenome era. Gigascience.

[38] Li M (2022). Trends in insulin resistance: insights into mechanisms and therapeutic strategy. Signal Transduct Target Ther.

[39] Vrooman LA, Bartolomei MS (2017). Can assisted reproductive technologies cause adult-onset disease? Evidence from human and mouse. Reprod Toxicol.

[40] Montjean D (2022). Impact of endocrine disruptors upon non-genetic inheritance. Int J Mol Sci.

[41] Davey RA, Grossmann M (2016). Androgen receptor structure, function and biology: from bench to bedside. Clin Biochem Rev.

[43] Willems M (2022). Transcriptomic differences between fibrotic and non-fibrotic testicular tissue reveal possible key players in Klinefelter syndrome-related testicular fibrosis. Sci Rep.

[44] Palmer NO (2012). Impact of obesity on male fertility, sperm function and molecular composition. Spermatogenesis.

[45] Finelli R (2021). Impact of alcohol consumption on male fertility potential: a narrative review. Int J Environ Res Public Health.

[46] Han G (2024). Ethanol-related transcriptomic changes in mouse testes. BMC Genomics.

[47] Dong J (2024). Metformin improves obesity-related oligoasthenospermia via regulating the expression of HSL in testis in mice. Eur J Pharmacol.

[48] Cescon M (2020). Environmental impact on male (In)Fertility via epigenetic route. J Clin Med.

[49] Kumar N, Singh AK (2022). Too advanced for assessment? Advanced materials, nanomedicine and the environment. Environ Sci Eur.

[50] Giudice FD (2022). The association of impaired semen quality and pregnancy rates in assisted reproduction technology cycles: Systematic review and meta-analysis. Andrologia.

[51] Garrido N (2023). Sperm epigenetics landscape: correlation with embryo quality, reproductive outcomes and offspring’s health. Panminerva Med.

[52] Keyhan S (2021). Male obesity impacts DNA methylation reprogramming in sperm. Clin Epigenetics.

[53] Chen S (2015). Corrigendum: Assisted reproduction causes placental maldevelopment and dysfunction linked to reduced fetal weight in mice. Sci Rep.

[54] Hayward CE (2016). Placental adaptation: what can we learn from birthweight:placental weight ratio?. Front Physiol.

[55] Nam H-K, Lee K-H (2018). Small for gestational age and obesity: epidemiology and general risks. Ann Pediatr Endocrinol Metab.

[56] Fahed G (2022). Metabolic syndrome: updates on pathophysiology and management in 2021. Int J Mol Sci.

[58] Bruner-Tran KL (2014). Developmental exposure of mice to dioxin promotes transgenerational testicular inflammation and an increased risk of preterm birth in unexposed mating partners. PLoS One.

[59] Takeda N (2016). Viable offspring obtained from Prm1-deficient sperm in mice. Sci Rep.

[60] Briley SM (2016). Reproductive age-associated fibrosis in the stroma of the mammalian ovary. Reproduction.

[61] Landini G (2021). Colour deconvolution: stain unmixing in histological imaging. Bioinformatics.

[62] Ruifrok AC, Johnston DA (2001). Quantification of histochemical staining by color deconvolution. Anal Quant Cytol Histol.

[63] Luense LJ (2019). Gcn5-mediated histone acetylation governs nucleosome dynamics in spermiogenesis. Dev Cell.

[64] Nakata H (2015). Quantitative analysis of the cellular composition in seminiferous tubules in normal and genetically modified infertile mice. J Histochem Cytochem.

[65] de Waal E (2014). In vitro culture increases the frequency of stochastic epigenetic errors at imprinted genes in placental tissues from mouse concepti produced through assisted reproductive technologies. Biol Reprod.

[66] Zhou W (2022). DNA methylation dynamics and dysregulation delineated by high-throughput profiling in the mouse. Cell Genom.

[67] Zhou W (2018). SeSAMe: reducing artifactual detection of DNA methylation by Infinium BeadChips in genomic deletions. Nucleic Acids Res.

