# Supplementary material for: In vitro fertilization induces reproductive changes in male mouse offspring and has multigenerational effects
Source: JCI Insight. 2025 Mar 4;10(8):e188931. doi: 10.1172/jci.insight.188931 (PMC12016927; doi:10.1172/jci.insight.188931)
Supplement: Supplemental data [file jciinsight-10-188931-s183.pdf]

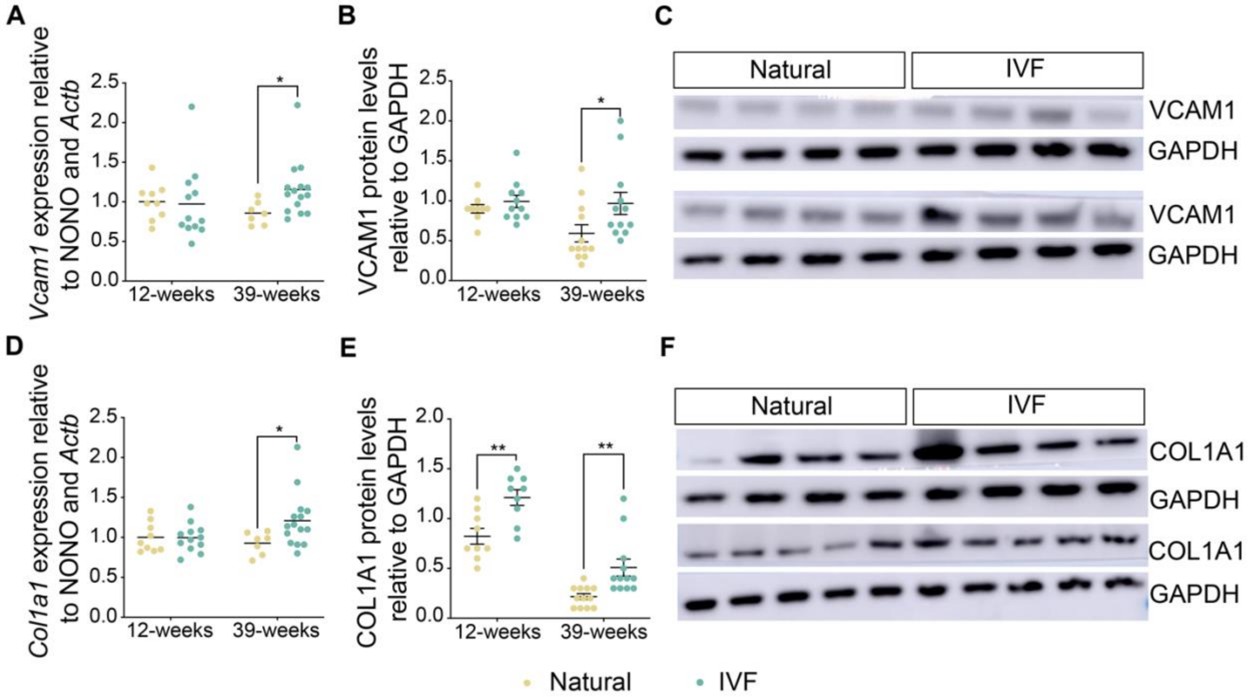

Supplemental Figure 1 (corresponding to main Figure 2). Gene expression and protein levels of *Col1a1* and *Vcam1*. Real time PCR using RNA from testis from 12- and 39-week-old offspring to measure expression of (A) *Vcam1*, and (D) *Col1a1* relative to *Nono* and *Actb*. Western blot for protein in whole testis for (B) VCAM1 and (E) COL1A1are relative to GAPDH, (C) and (F) western blots images corresponding to each protein. Data are depicted as mean±s.e.m, *n*=10-15 per group. The black line represents the mean of each group. Statistical significance was determined by t- test, *P<0.05, and **P<0.01 when compared groups against Natural.

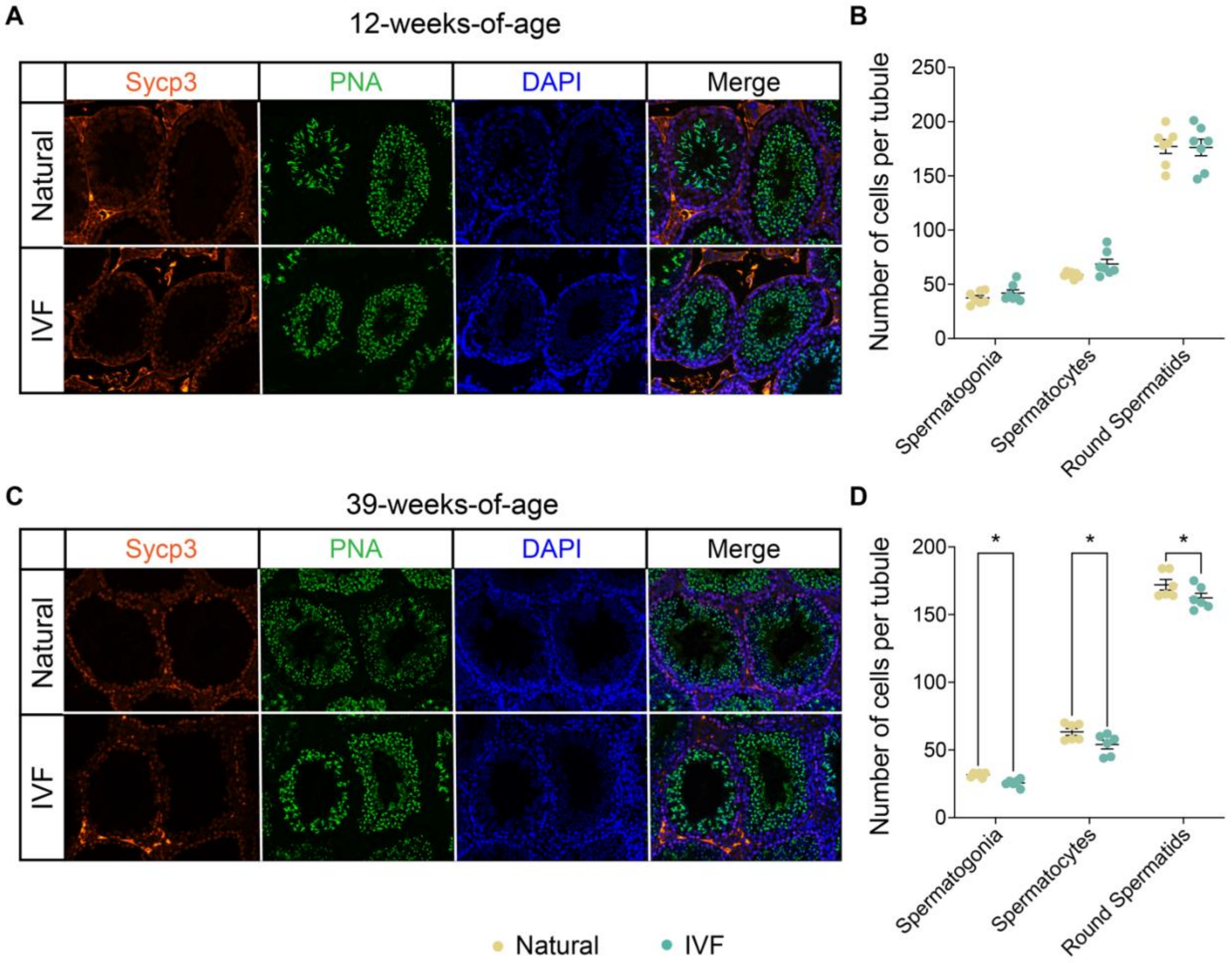

Supplemental Figure 2 (corresponding to main Figure 2). Average number of spermatogonia, spermatocytes and round spermatids in stage II-III and stage VI-VIII combined seminiferous tubules of Natural and IVF testes at 12 weeks (A) through immunohistochemistry of whole testis, and (B) by direct counting. Similar assessments were made at 39 weeks using (C) immunohistochemistry and (D) direct counting. Spermatogonia and spermatocytes were quantified following immunofluorescence staining of testes sections with Anti-Sycp3, secondary Alexa Fluor 647and DAPI. Round spermatids were counted following immunofluorescence staining of testes sections with PNA-488 to acrosome staining and DAPI. Data are depicted as mean±s.e.m, *n*=6 per group. The black line represents the mean of each group. Statistical significance was determined by t- test, *P<0.05 when compared groups against Natural.

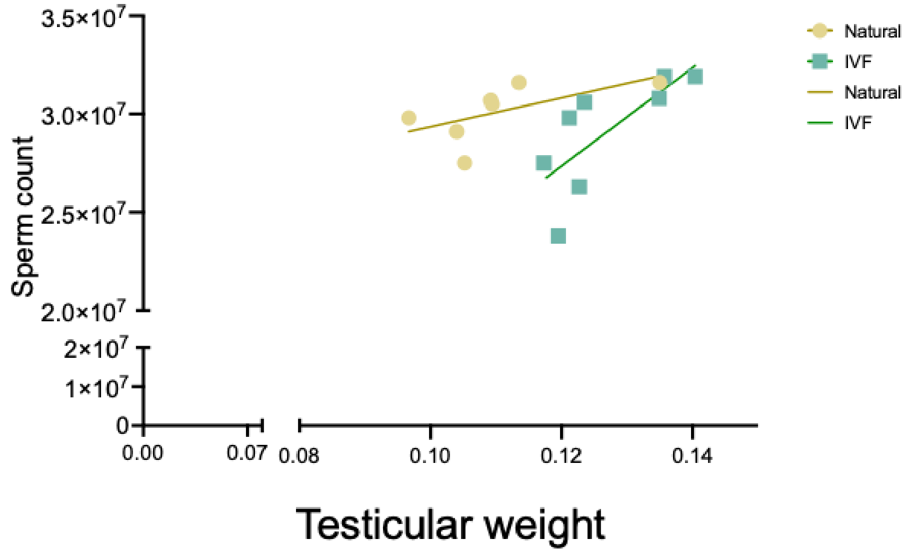

Supplemental Figure 3 (corresponding to main Figure 3). Correlation between sperm count and testicular weight in both IVF and Natural offspring.

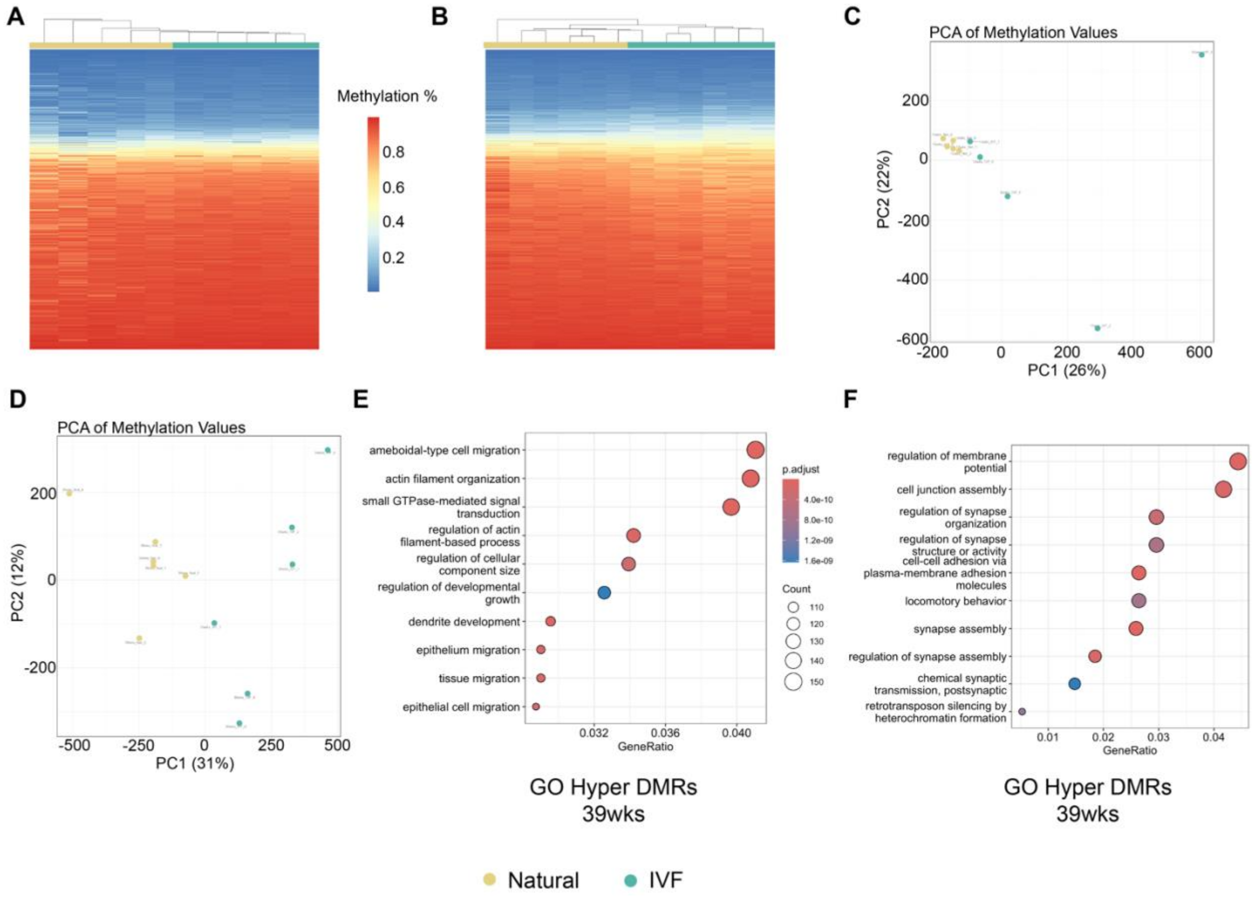

Supplemental Figure 4 (corresponding to main Figure 3). Heatmap of all the probes from the Illumina Beadchip array using sperm: (A) 12-weeks-of-age, and (B) 39-weeks-of-age. Principal component analysis (PCA) for sperm samples run in the Illumina Beadchip array: (C) 12-weeks-of-age, and (D) 39-weeks-of-age. Gene ontology (GO) analysis at 39-weeks-of-age: (E) GO with Hyper DMRs, and (F) GO with Hypo DMRs.

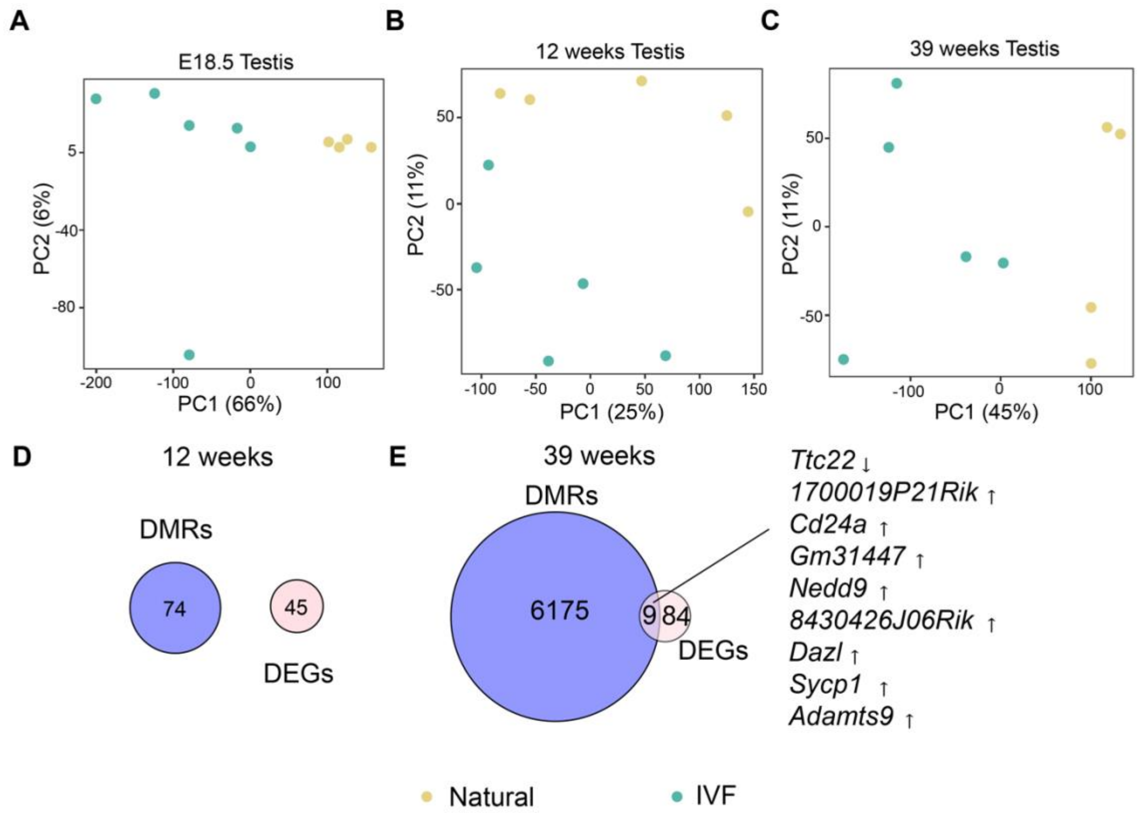

Supplemental Figure 5 (corresponding to main Figure 4). Principal component analysis (PCA) for RNAseq results. PCA before DEG analysis for (A) E18.5 Testis, (B) 12 weeks Testis, (C) 39 weeks Testis. Overlap between sperm DMRs and DEGs testis: (D) 12 weeks Testis and (E) 39 weeks Testis.

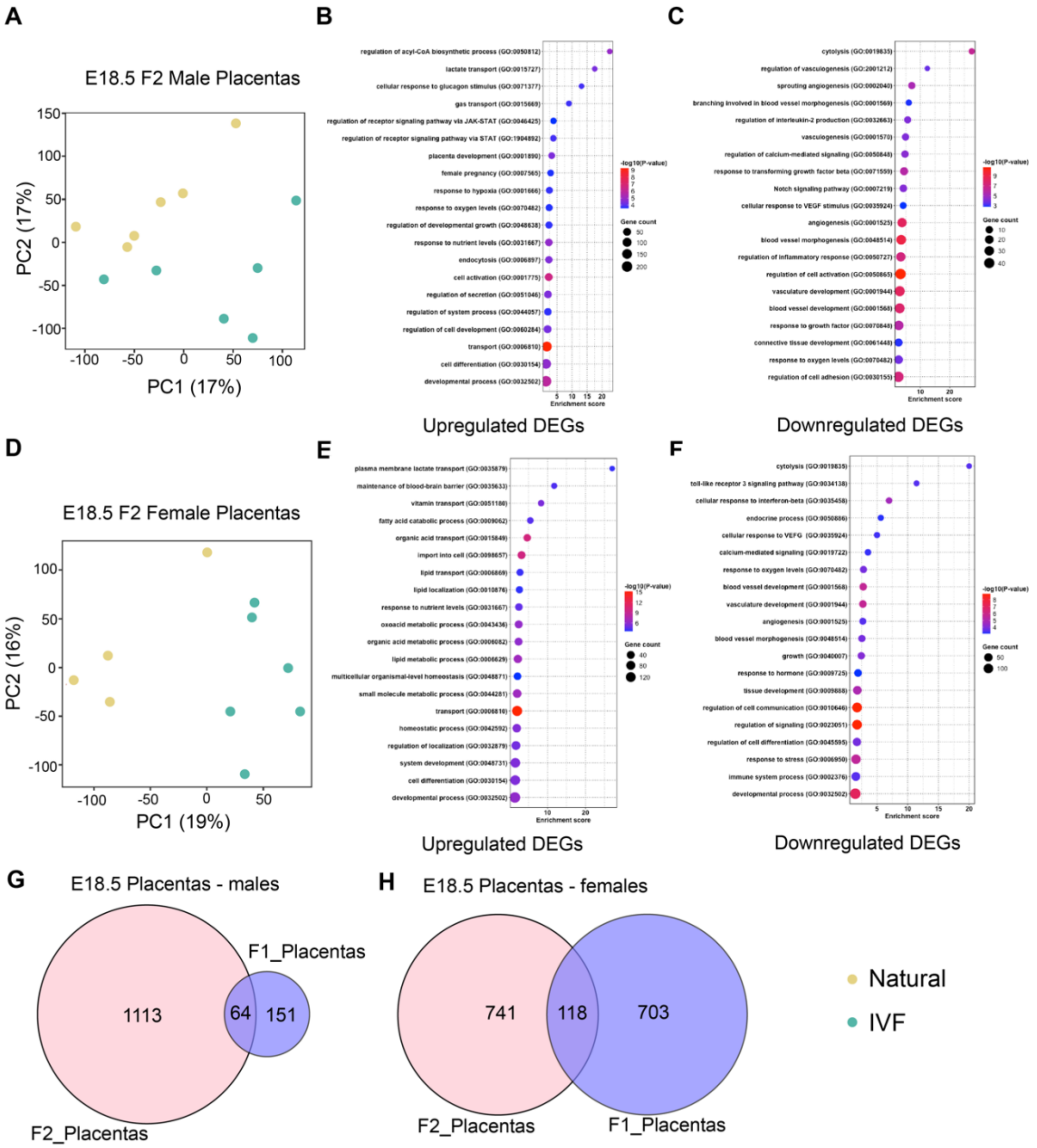

Supplemental Figure 6 (corresponding to main Figure 6). Principal component analysis (PCA) for RNAseq results: (A) PCA before DEG analysis for E18.5 F2 male placentas, gene ontology analysis for (B) upregulated genes in F2 male placentas, and (C) downregulated genes in F2 male placentas. (D) PCA before DEG analysis for E18.5 F2 female placentas, gene ontology analysis for (E) upregulated genes in F2 female placentas, and (F) downregulated genes in F2 female placentas. Overlap between F1 DEGs and F2 DEGs placentas: (G) E18.5 males and (H) E18.5 females.

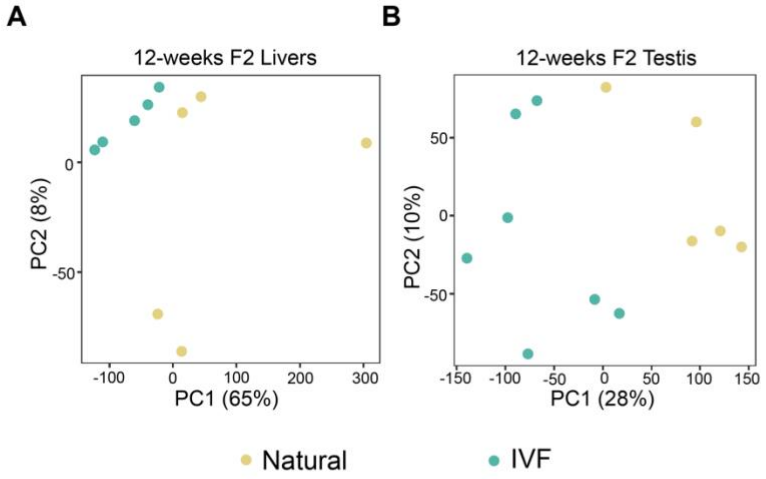

Supplemental Figure 7 (corresponding to main Figure 6). Principal component analysis (PCA) for RNAseq results. PCA before DEG analysis for male second generation (A) livers and (B) testis.

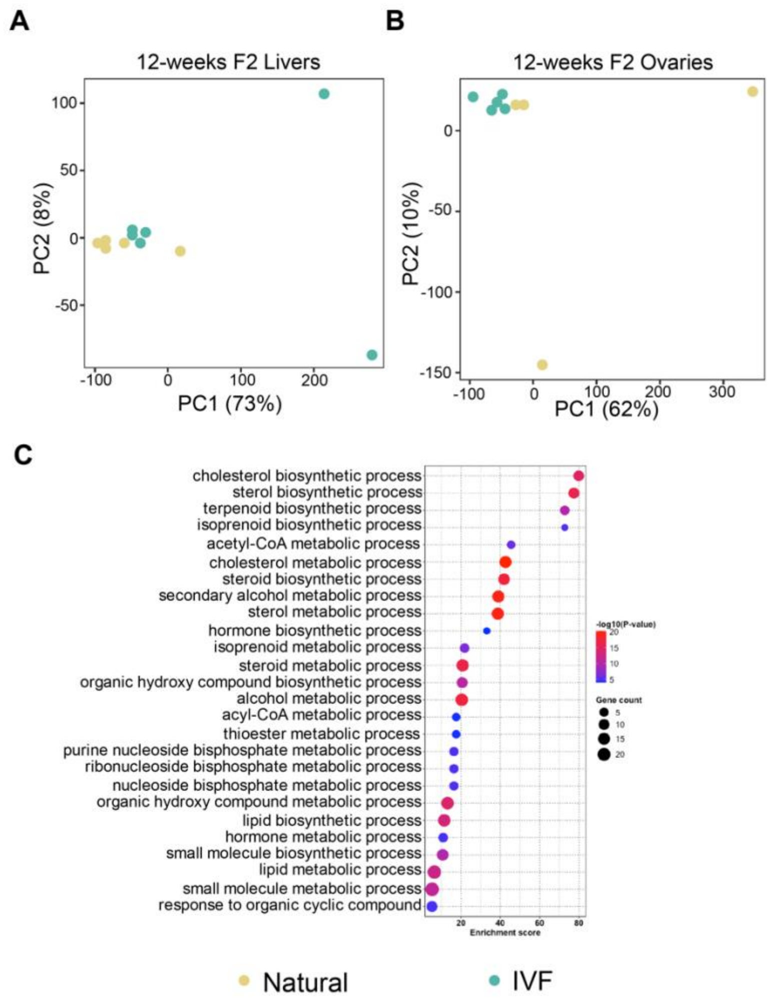

Supplemental Figure 8 (corresponding to main Figure 7). Principal component analysis (PCA) for RNAseq results. PCA before DEG analysis for female second generation (A) livers and (B) ovaries. (C) Gene ontology analysis for upregulated genes in ovaries from second generation females.

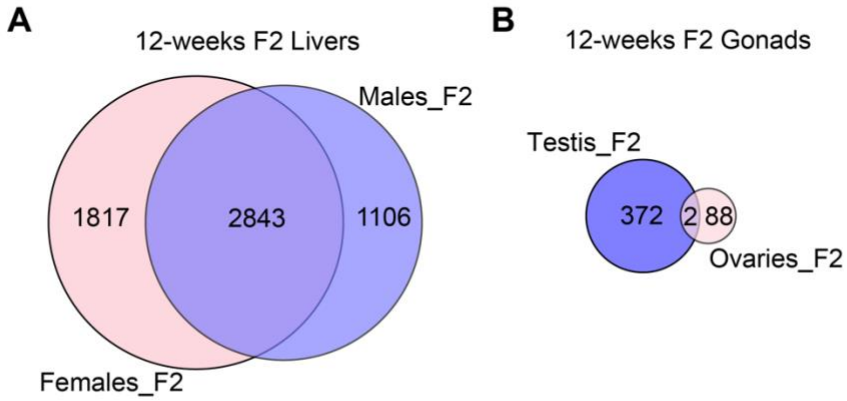

Supplemental Figure 9 (corresponding to main Figure 6 and 7). Venn diagrams showing common DEGs males and females for (A) livers and (B) gonads.

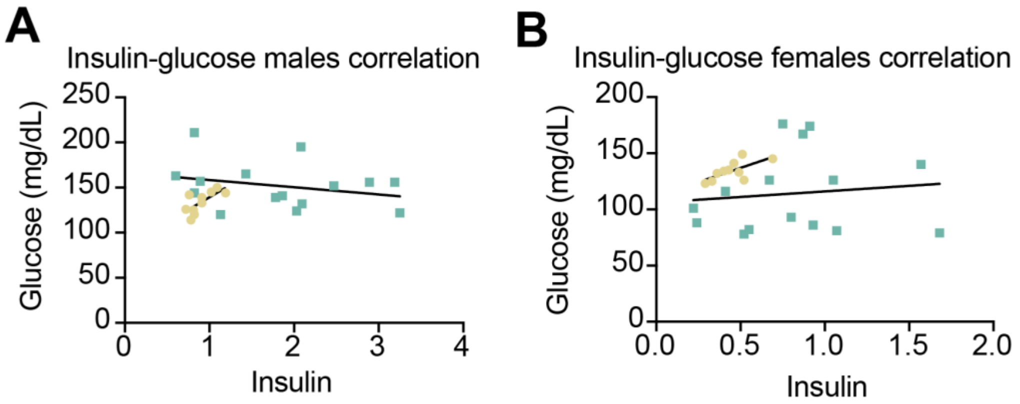

	Insulin vs Glucose Nat	Insulin vs Glucose IVF
Pearson r	0.7117	-0.2733
P (two-tailed)	0.021	0.3244
R squared	0.5065	0.0746

	Insulin vs Glucose Nat	Insulin vs Glucose IVF
Pearson r	0.6764	0.1167
P (two-tailed)	0.0317	0.6788
R squared	0.4575	0.0136

Natural IVF

Supplemental Figure 10 (corresponding to main Figure 6 and 7). Correlation analysis between glucose and insulin in offspring from IVF or Natural (A) males or (B) females.
