# Supplementary material for: In vitro fertilization induces reproductive changes in male mouse offspring and has multigenerational effects
Source: JCI Insight. 2025 Mar 4;10(8):e188931. doi: 10.1172/jci.insight.188931 (PMC12016927; doi:10.1172/jci.insight.188931)
Supplement: Unedited blot and gel images [file jciinsight-10-188931-s184.pdf]

Full unedited gel for Figure 1: Androgen Receptor Antibody (see methods for more information)

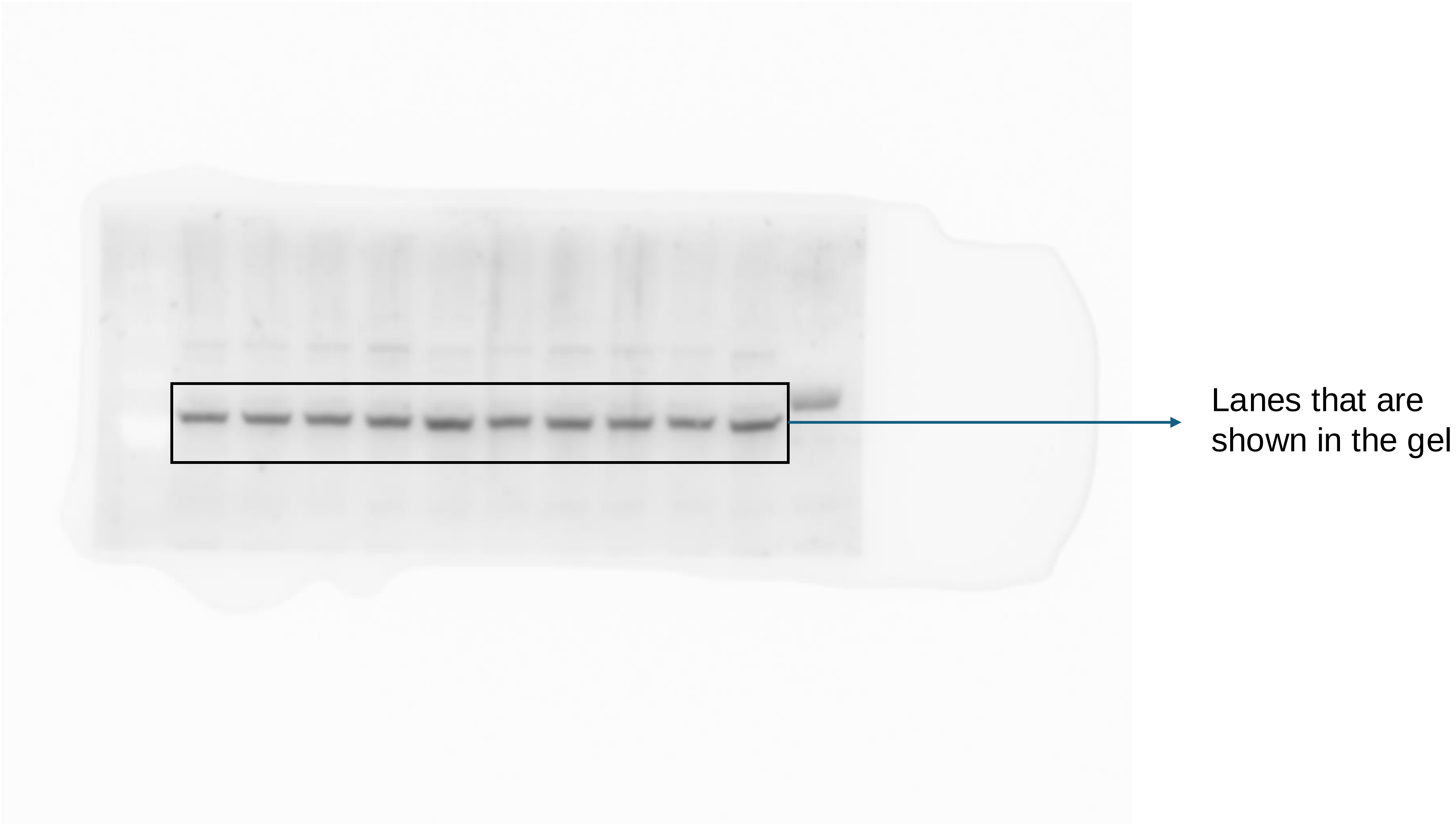

Full unedited gel for Figure 1: Anti-GADPH Antibody (see methods for more information)

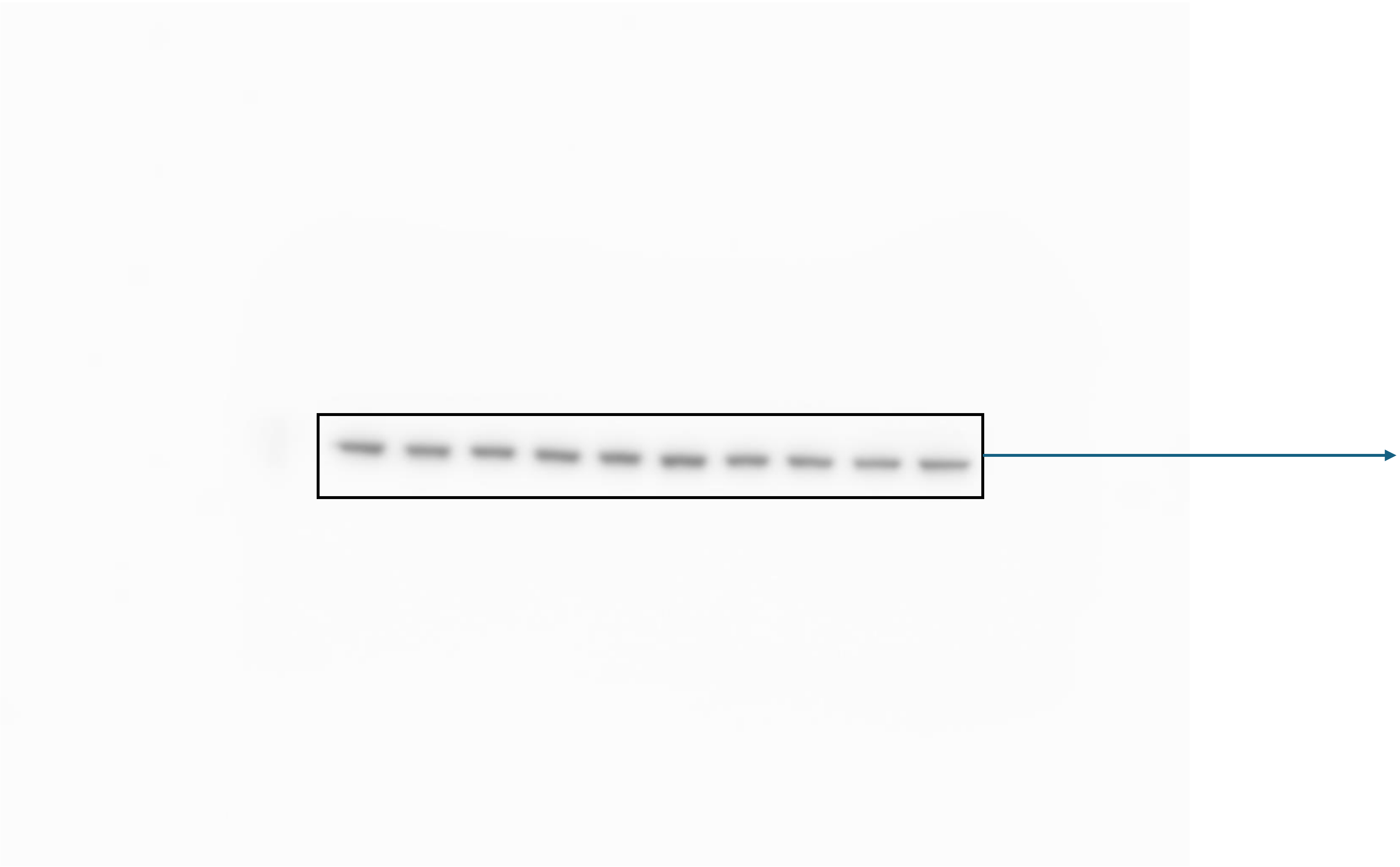

Lanes that are shown in the gel

Full unedited gel for Supplemental Figure 1C: Anti-VCAM1 Antibody (see methods for more information) – Upper panel

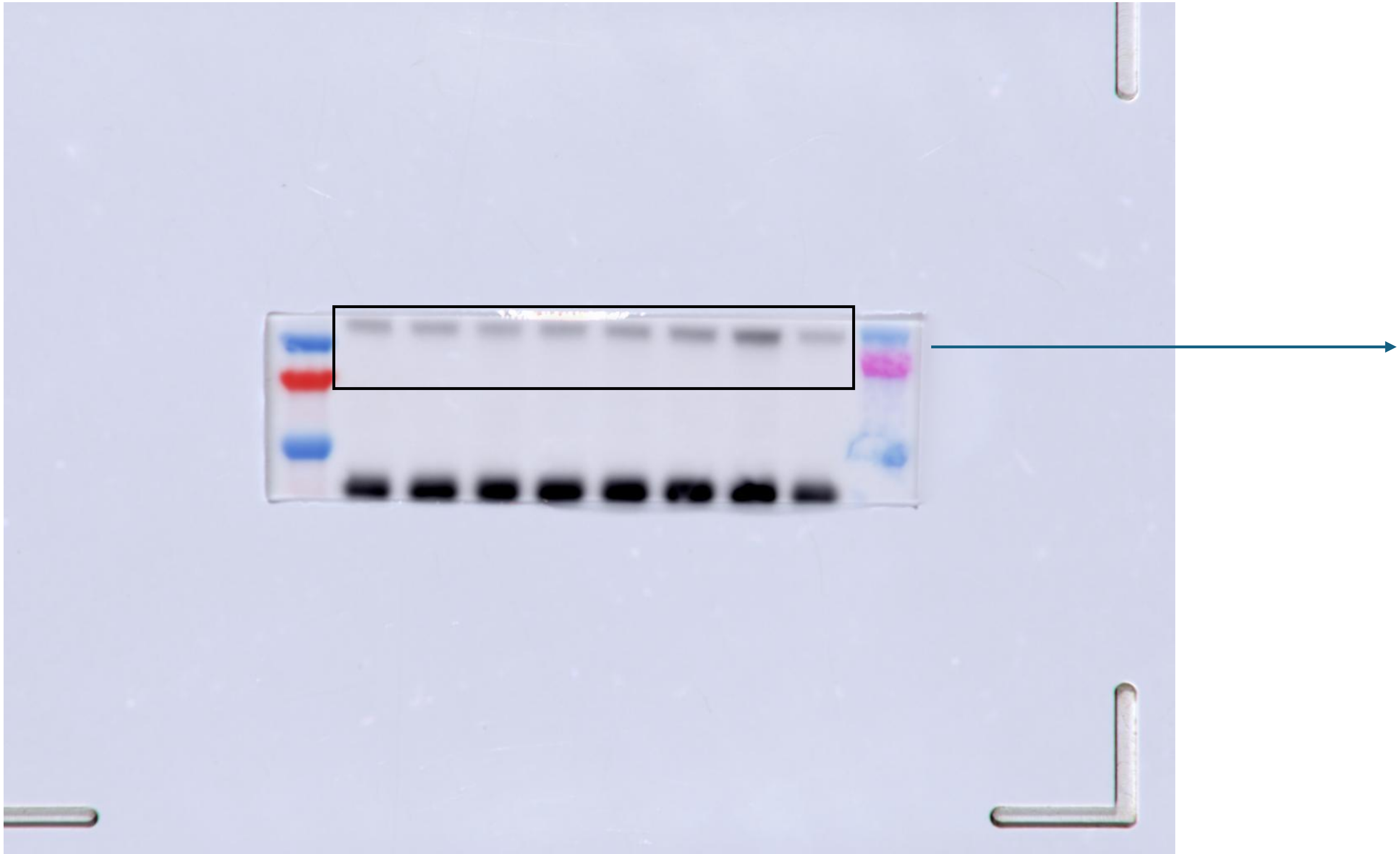

Lanes that are shown in the gel

Full unedited gel for Supplemental Figure 1C: Anti-GAPDH Antibody (see methods for more information) Upper panel

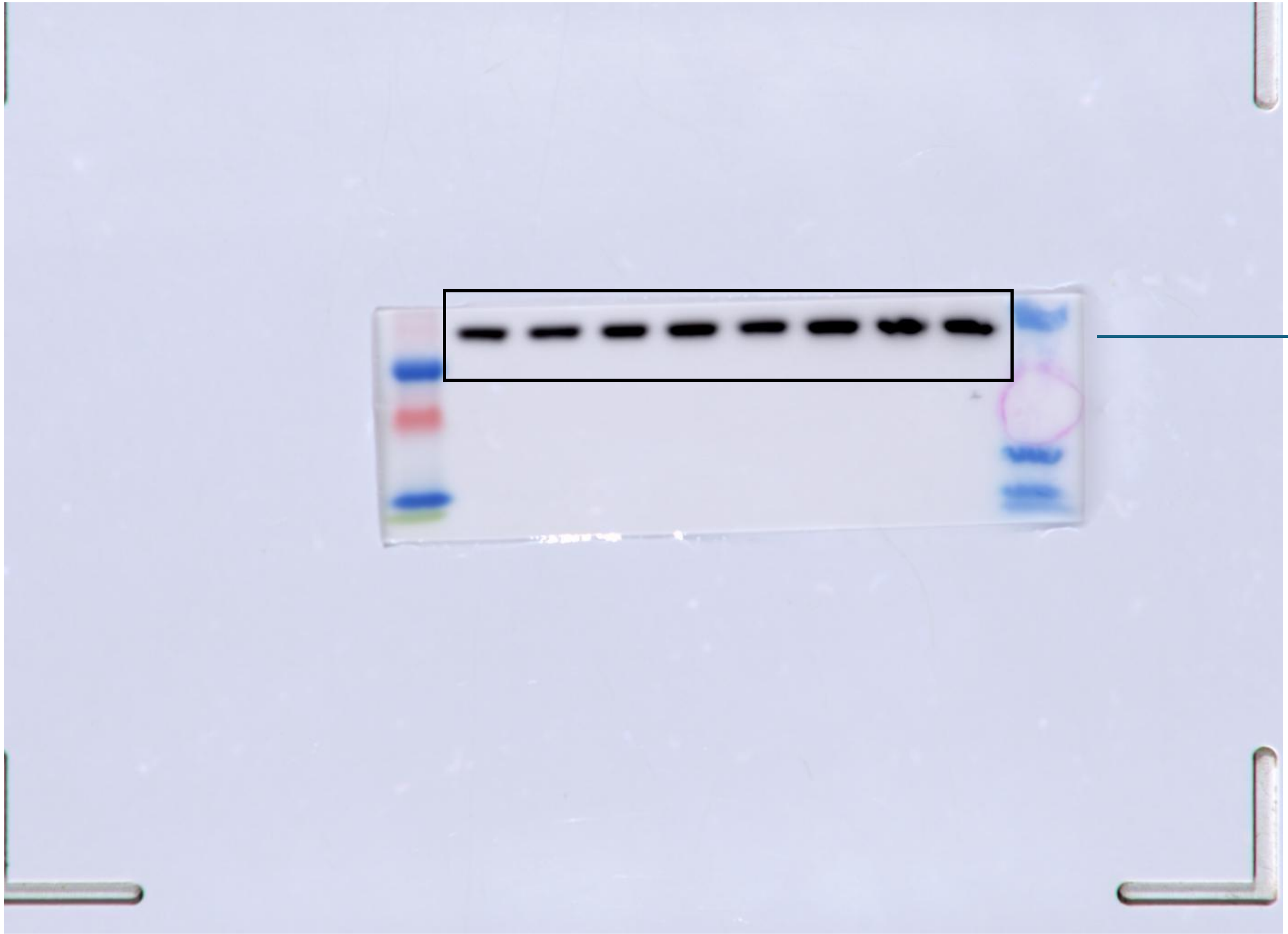

Lanes that are shown in the gel

Full unedited gel for Supplemental Figure 1C: Anti-VCAM1 Antibody (see methods for more information) – Lower panel

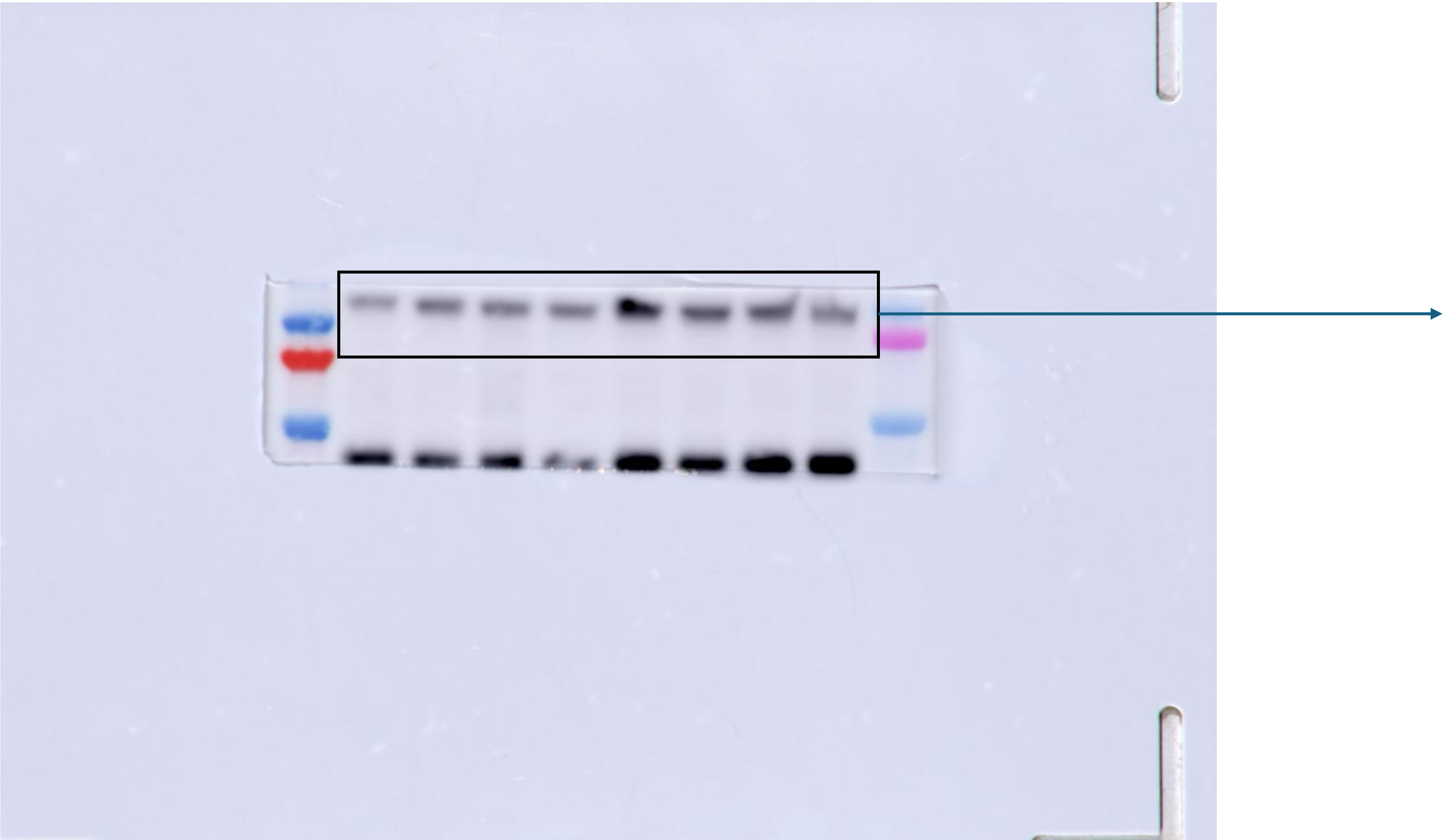

Lanes that are shown in the gel

Full unedited gel for Supplemental Figure 1C: Anti-GAPDH Antibody (see methods for more information) Lower panel

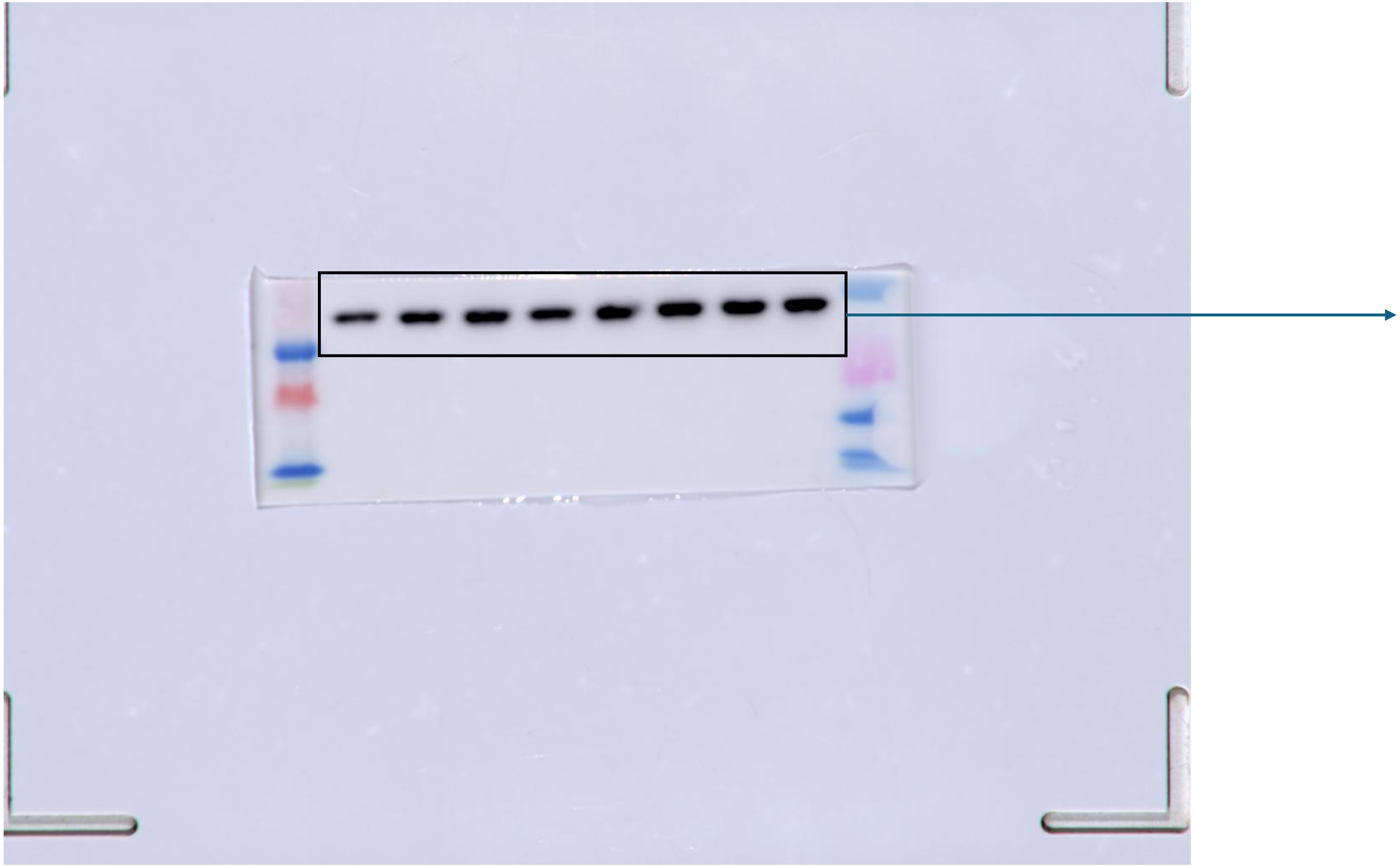

Lanes that are shown in the gel

Full unedited gel for Supplemental Figure 1F: Anti-COL1A1 Antibody (see methods for more information) – Upper panel

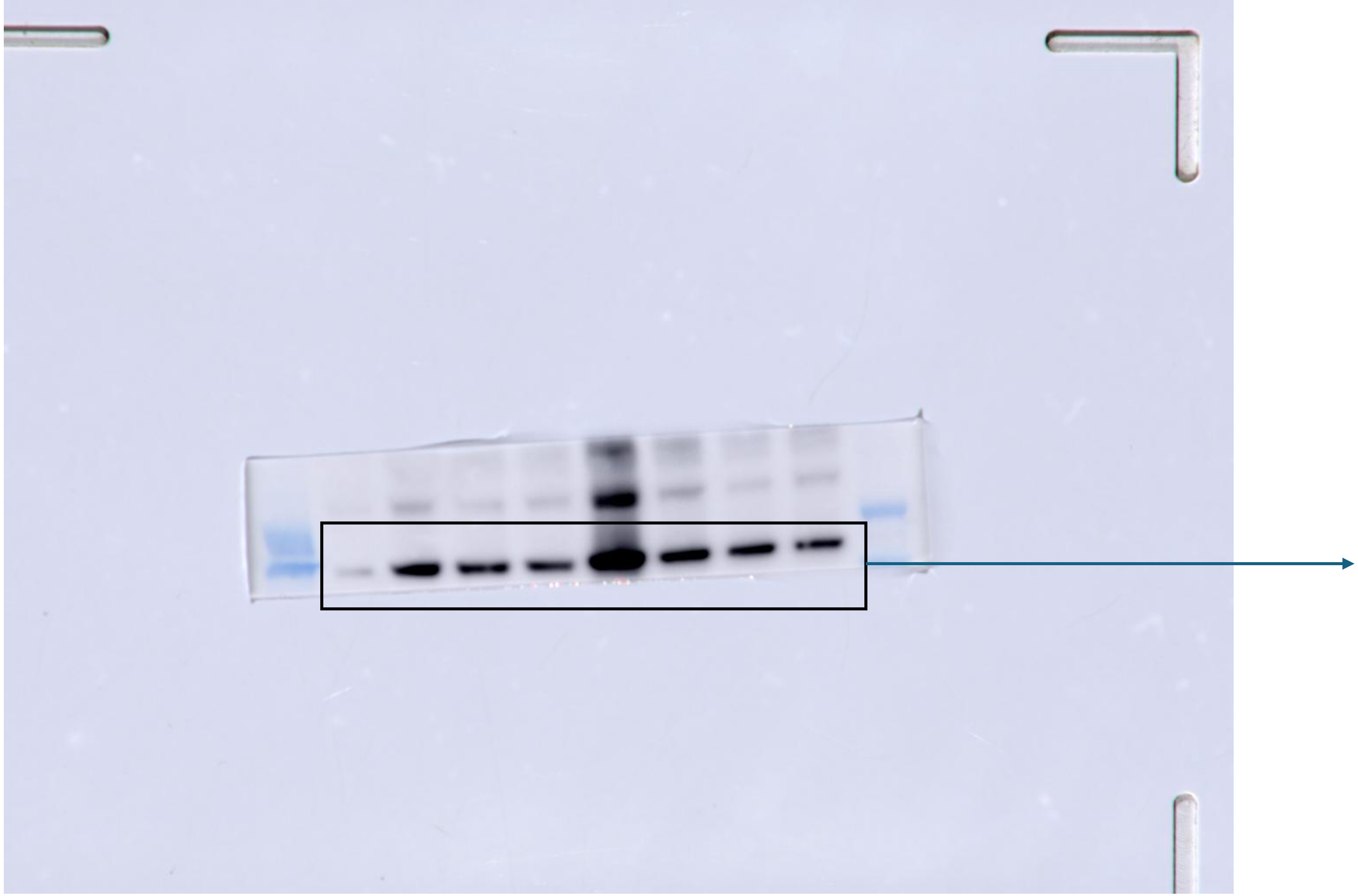

Lanes that are shown in the gel

Full unedited gel for Supplemental Figure 1F: Anti-GAPDH Antibody (see methods for more information) Upper panel

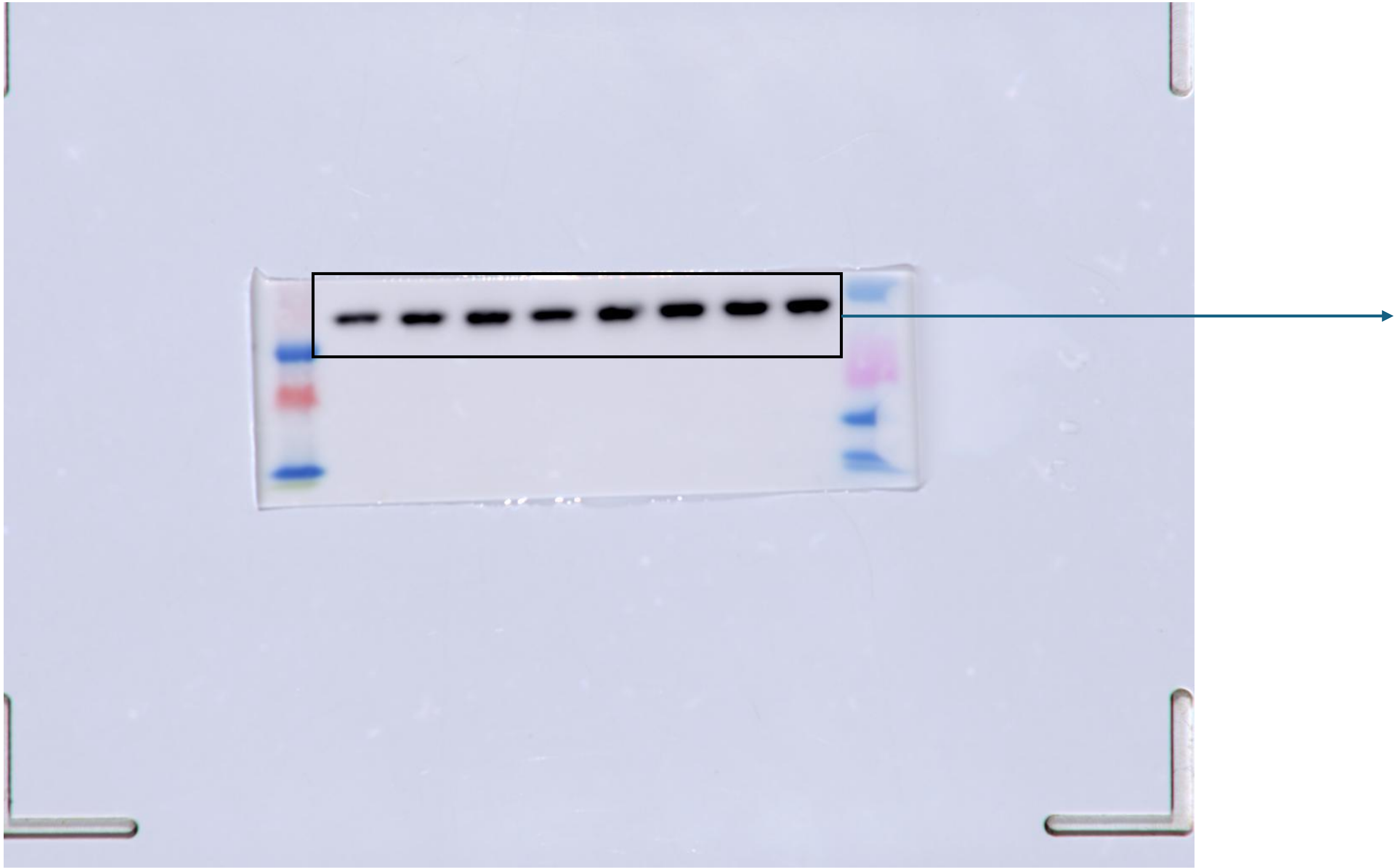

Lanes that are shown in the gel
